# Preventing COVID-19 outbreaks through surveillance testing in healthcare facilities: a modelling study

**DOI:** 10.1186/s12879-022-07075-1

**Published:** 2022-01-29

**Authors:** Tim Litwin, Jens Timmer, Mathias Berger, Andreas Wahl-Kordon, Matthias J. Müller, Clemens Kreutz

**Affiliations:** 1 Institute of Medical Biometry and Statistics (IMBI), Faculty of Medicine and Medical Center, University of Freiburg, 79104 Freiburg, Germany; 2 Freiburg Center for Data Analysis and Modelling (FDM), University of Freiburg, 79104 Freiburg, Germany; 3 Institute of Physics, University of Freiburg, 79104 Freiburg, Germany; 4 Centre for Integrative Biological Signalling Studies (CIBSS), University of Freiburg, 79104 Freiburg, Germany; 5 Department of Psychiatry and Psychotherapy, Medical Center, Faculty of Medicine, University of Freiburg, 79104 Freiburg, Germany; 6 Oberberg Hospital Schwarzwald, 78132 Hornberg, Germany; 7 Oberberg Group, 10117 Berlin, Germany; 8 Faculty of Medicine, Justus-Liebig-University Giessen, 35392 Giessen, Germany

**Keywords:** Infectious disease surveillance, COVID-19, Long-term care, Point-of-care testing, Agent-based model

## Abstract

**Background:**

Surveillance testing within healthcare facilities provides an opportunity to prevent severe outbreaks of coronavirus disease 2019 (COVID-19). However, the quantitative impact of different available surveillance strategies and their potential to decrease the frequency of outbreaks are not well-understood.

**Methods:**

We establish an individual-based model representative of a mental health hospital yielding generalizable results. Attributes and features of this facility were derived from a prototypical hospital, which provides psychiatric, psychosomatic and psychotherapeutic treatment. We estimate the relative reduction of outbreak probability for three test strategies (entry test, once-weekly test and twice-weekly test) relative to a symptom-based baseline strategy. Based on our findings, we propose determinants of successful surveillance measures.

**Results:**

Entry Testing reduced the outbreak probability by 26%, additionally testing once or twice weekly reduced the outbreak probability by 49% or 67% respectively. We found that fast diagnostic test results and adequate compliance of the clinic population are mandatory for conducting effective surveillance. The robustness of these results towards uncertainties is demonstrated via comprehensive sensitivity analyses.

**Conclusions:**

We conclude that active testing in mental health hospitals and similar facilities considerably reduces the number of COVID-19 outbreaks compared to symptom-based surveillance only.

**Supplementary Information:**

The online version contains supplementary material available at 10.1186/s12879-022-07075-1.

## Background

Treatment and care facilities with intermediate to long-term treatment durations pose a setting in which active COVID-19 surveillance strategies are urgently required. This became apparent from reports of disastrous outbreaks in skilled nursing facilities [1] which host a population at an age-related high risk of fatal disease courses [2]. Psychiatric and psychosomatic facilities are faced with the challenge to maintain and ensure patient and staff security with at the same time increasing psychiatric symptoms in patients [3] and safety concerns in regard to a possible hospital stay. Establishing ways to effectively protect these populations allows these facilities to continue their regular functions despite of the current circumstances. Thus, these facilities in particular may substantially benefit from interventions intended to decrease the risk of possible COVID-19 outbreaks.

Surveillance aims at the disruption of evolving infection clusters in order to halt the spread of infection. Existing COVID-19 surveillance strategies revolve around detection of infected individuals in order to isolate them and their close contacts. To this end, possible infections are commonly confirmed by polymerase chain reaction (PCR) tests [4]. Yet, such tests are subject to certain structural constraints as they need to be performed in a laboratory which effectively delays the initiation of isolation and tracing. To circumvent these constraints, the use of point-of-care (PoC) tests in the form of faster but less sensitive antigen tests has been proposed. Although those tests might only detect individuals with high enough viral loads to actually be infectious [5], the fact that those tests can be carried out by each individual autonomously allows to make at least a tentative COVID-19 diagnosis with greater flexibility and in a less centralized manner. This highlights their possible use for surveillance in single institutions.

High quality evidence about the benefits of surveillance testing is sparse. Mathematical modelling studies complement empirical evidence by conceptualizing the important aspects of the problem [6] and by identifying the main determinants responsible for observed phenomena. There are existing modelling studies that tackle different aspects of surveillance testing, such as limited availability of test resources [7], cohorting of staff and residents [8], and regular testing of staff/residents at different frequencies [9]. Those results are of limited generalizability: Most models report outcomes for which a reasonable quantification of uncertainties is impossible, e.g. the cumulative number of infections at a late stage of the outbreak. Uncertainties about the structure of the infection spread and the underlying epidemiological parameters amplify the uncertainties of the outcome in a non-linear manner which complicates the conduct of rigorous analyses.

We add to the available body of evidence by proposing an individual-based model representative of a prototypical psychiatric-psychosomatic hospital offering treatment to patients for extended periods of time. Our study aims at providing some generalizable key results with a robust quantification of their uncertainty. This is achieved by conducting a comprehensive set of sensitivity analyses of parameters and various structural assumptions presented in an easily accessible form.

## Methods

### Structure of simulation model

**Fig. 1:**
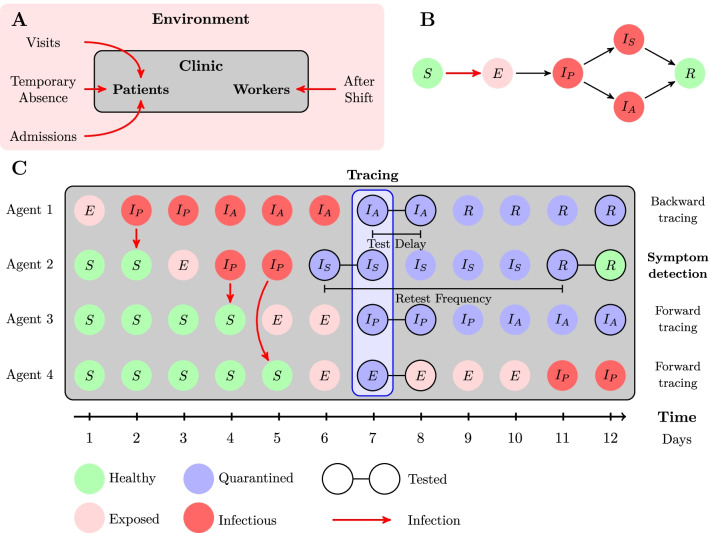
Schematic illustration of virus intrusion (**A**), disease progression (**B**) and the implementation of surveillance (**C**). $S$: Susceptible, $E$: Exposed, ${I}_{P}$: Presymptomatically Infectious, ${I}_{S}$: Symptomatically Infectious, ${I}_{A}$: Asysmptomatically Infectious, $R$: Recovered. Panel **C** demonstrates symptom-based baseline surveillance in a hypothetical case scenario. Agent 1 has been infected outside of the clinic (index case) and infects agent 2, who goes on to infect agent 3 and 4. On day 6, agent 2 is isolated due to developing symptoms and once the case is ascertained a day later, contact tracing isolates the primary infector (backward tracing) and subsequent infections by agent 2 (forward tracing). The isolated individuals are then tested, confirming that agent 1 and agent 3 are infectious. Agent 4 is released as the infection is not yet detectable

We simulated the propagation of infection and implementation of surveillance measures in a hospital (Oberberg Fachklinik Schwarzwald) with 60 inpatients with an average duration of stay of 8 weeks and 80 staff members. The hypothetical clinics were initialized with fully susceptible populations whose dynamic evolutions were simulated in daily increments for a total simulation time of 100 days. The facility was modelled as a semi-closed environment: Propagation of infection within the clinic was treated as a closed system, but interactions with the environment outside of the clinic could introduce infected individuals into the clinic. Figure 1A shows the different possibilities of virus intrusion into the clinic: New infectious patients may be admitted to the clinic, patients may be visited by infectious visitors or be infected on a temporary weekend leave from the clinic while staff could get infected between work shifts. Each simulation was subject to a set of parameter assumptions; the sets of parameters used in this study were extracted from the literature and are summarized in Table 1. A detailed discussion of epidemiological parameters and their implications for the model is provided in Additional file 1.

**Table 1:** Summary of the used model parameters and their uncertainties according to literature

Name	Unit	Lower	Best	Upper	Description	Source
Asymptomatic fraction	[%]	10	20	30	Fraction of asymptomatic disease courses	[10–13]
Asymptomatic infectivity	[%]	40	70	100	Infectiousness of asymptomatic individuals compared to symptomatic individuals	[1, 11, 12]
False symptoms	[agents/day]	0.5	1	2	Average daily amount of non-COVID-19 related symptomatic individuals	Assumed
False traces	[agents]	4	8	12	Average amount of erroneously traced individuals assuming perfect tracing efficiency	Assumed
Heterogeneity modifier	[]	2	4	6	Scaling factor of infectivity in transmission matrix of high-risk/low-risk staff	Assumed
Incubation mean	[days]	5	5.5	6	Mean of incubation time	[14, 15]
Incubation SD	[days]	2.1	2.3	2.5	Standard deviation of incubation time	[14]
Infectivity heterogeneity	[]	1	1.5	1000	Heterogeneity in individual infectivity: Shape parameter of the Gamma-distribution	Derived from [16, 17]
Isolation fraction	[%/day]	50	70	90	Fraction of symptomatic individuals isolated daily	Assumed
Outside infection	[]	0.01	0.04	0.16	Scaling factor of infection risk outside of clinic	Assumed
Peak infectiousness	[days]	− 1	1	3	Time shift of peak infectiousness relative to symptom onset	[18]
Prevalence	[%]	0.005	0.02	0.08	COVID-19 prevalence in population including non-confirmed cases	Assumed
R0	[]	1.5	3	5	Average number of infections an individual causes inside of clinic	Assumed
Symptom mean	[days]	3.5	5	6.5	Mean of symptomatic infectious time	Derived from [19–22]
Symptom SD	[days]	1.1	1.5	1.9	Standard deviation of symptomatic infectious time	Derived from [19–22]
Test compliance	[%]	60	80	100	Fraction of individuals compliant with repeated surveillance testing	Assumed
Test sensitivity	[%]	80	90	100	Sensitivity of diagnostic test	Derived from [5, 23, 24]
Test specificity	[%]	98	99.5	100	Specificity of diagnostic test	Assumed
Tracing fraction	[%]	50	70	90	Fraction of infections reconstructed by contact tracing	Assumed

Upper and lower bounds are used for 1-way sensitivity analysis as they represent the existing lack of knowledge about these parameters. The term “derived from” indicates that input from the stated sources was not directly applicable in the model and required some form of subjective judgement and modification prior to the inclusion into the model

### Modelling infection spread

The infection dynamics were implemented in the model at the level of individual agents which represented individuals in the clinic population. Individual-based models offer a high flexibility and allow for incorporation of inter-individual heterogeneity and inherent stochasticity, such that they are well suited to model relevant features of the epidemiological dynamics realistically [25]. The current state of disease progression was tracked for every agent individually. Based on the current state of the infected individuals within the clinic, the probability of infection was derived for all susceptible individuals. The risk of infection depended on the disease states of the agents but also on various dynamic properties, such as quarantine, possible absence from the clinic and current infectivity of agents. The infection dynamics were stochastic, i.e. it was randomly drawn whether an agent was infected on a given day or not.

The disease progression was modelled structurally similar to a homogeneous stochastic SEIR-model [26], but extended to incorporate characteristic features of COVID-19 and a more realistic transmission structure. Asymptomatic individuals who display no noticeable symptoms nevertheless show a significant transmission potential [12]. This is partly due to presymptomatic transmission, as viral shedding begins already before symptom onset [27], i.e. during the incubation period. This lead to an extended list of states incorporated in our model: Susceptible $\mathrm{S}$, Exposed $\mathrm{E}$, Presymptomatically Infected ${\mathrm{I}}_{\mathrm{P}}$, Symptomatically Infected ${\mathrm{I}}_{\mathrm{S}}$, Asymptomatically Infected ${\mathrm{I}}_{\mathrm{A}}$, Removed $\mathrm{R}$. The progression of disease states is illustrated in Fig. 1B.

**Fig. 2:**
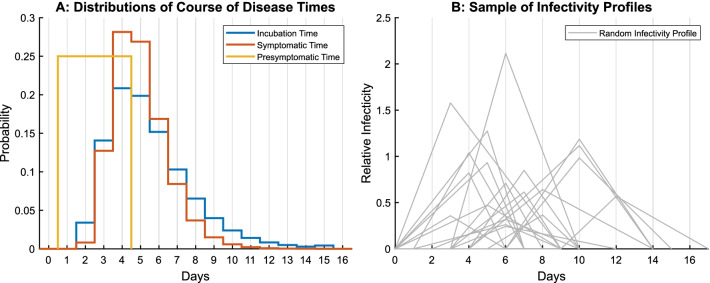
Illustration of randomized disease state retention times (**A**) and random sample of 20 infectivity profiles (**B**). Distributions of retention times for different disease states (**A**) correspond to the best guess parameters extracted from literature. Each infectivity profile (**B**) describes the time course of infectiousness of one random individual. The individual profiles differ in their onsets and infectivity levels

Variations in the natural history of the disease are not only manifested in the display of symptoms but also in the timing of the different disease states. It has been shown that accounting for stochasticity in these timings significantly affects the modelled spread of a virus [26]. Consequently, the incubation time, the symptomatic time as well as the presymptomatic time were defined as random variables. The resulting distributions of these durations are visualized in Fig. 2A assuming best guess parameters. The range of the uniformly distributed presymptomatic time was based on [1, 18, 19, 27], incubation time and symptomatic time were lognormal with parameters as defined in Table 1. Additionally, individuals vary in their individual infectiousness due to their different biological and behavioural components [28]. Empirically, this varying degree of infectiousness can be used to explain the offspring distribution*,* i.e. the number of secondary cases caused by the respective primary cases [29]. For transmissions of SARS-CoV-2, these offspring distributions have been observed to be highly asymmetric [16, 17], implying the existence of super-spreading. Based on these observed distributions, similar distributions have been reproduced qualitatively in our model by defining the individual infectiousness to be Gamma-distributed, refer to Additional file 2 for details.

Temporal variations of the infectiousness of agents at different stages of their disease were represented by time-dependent infectivity profiles [28]. The infectivity of agents was modelled to increase linearly until a peak infectivity is reached, after which it will decrease linearly until the end of the symptomatic phase [30]. Figure 2B shows a sample of infectivity profiles for 20 random individuals. The profiles vary in their behaviour over time and in their general scale, representing random time spans for disease courses and agents with different transmission potential.

Heterogeneity in the transmission structure has been found to play a key role in infection spread, e.g. by application of infection models on networks [31] or age-stratified contact matrices [32]. In order to implement a relevant heterogeneous transmission structure into our model, the four agent classes—patients, low-risk staff, average-risk staff and high-risk staff—were defined. The transmission rates between these classes were modified using transmission matrices, which included information about the staff’s occupation and the duration of shifts. In order to adjust the scale of transmission dynamics, model parameters were calibrated to return pre-specified values of the reproduction number R0. A more detailed description of the modelled infection dynamics and the calibration process is provided in Additional file 2.

### Modelling conduct of surveillance

Surveillance measures are implemented to detect and prevent the spread of infection within the clinic. This is achieved by enforcing strict quarantine on individuals who display symptoms or individuals who were tested positive in screening measures, e.g. achieved by relocating the agent out of the clinic. Once an agent is quarantined, the agent was assumed to be non-infectious during the time of isolation. While this is likely not entirely realistic, this is a decent approximation in the light of the multitude of other, more critical modelling assumptions. The fundamental concepts of outbreak detection and containment represented a symptom-based baseline surveillance scenario which was common to all considered surveillance strategies. It comprised (i) isolation based on symptoms, (ii) case ascertainment via diagnostic testing, and (iii) subsequent contact tracing to isolate contacts at high risk of possible contagion due to previous contact with the index case.

Isolation based on symptoms was modelled as a daily probability of isolating an agent in the symptomatic state ${\mathrm{I}}_{\mathrm{S}}$. Typical symptoms of COVID-19 are not specific to this disease and may be mild, such that the possibility of unjustified isolations of healthy individuals has been included. In order to properly deal with such ambiguous cases, a case-ascertainment process is needed to distinguish non-infectious from infectious individuals. Modelling case-ascertainment required specification of a diagnostic test which could assess the disease state of an individual or, more specifically, whether the individual was infectious. The performance of the diagnostic test used in the model has been uniquely specified by its sensitivity, specificity, and test-to-result delay. Performance of the PoC test used in the model was based on the Panbio™ COVID-19 antigen rapid test (Abbott) [24] as employed in the factual hospital. Finally, contact tracing disrupts possible chains of infection once infected individuals are detected. It was modelled by immediate isolation of secondarily infected individuals once the primary case has been ascertained, given a certain success probability of locating the secondary cases. Figure 1C summarizes the three mentioned concepts inherent to baseline surveillance by displaying hypothetical chains of infection and their subsequent detection via the surveillance measure.

Four different surveillance strategies were evaluated quantitatively: The aforementioned symptom-based baseline surveillance strategy in which testing is only initiated because of symptoms and three active surveillance strategies. Active surveillance strategies comprised preventive testing of the clinic population. The first investigated strategy was the symptom-based baseline surveillance. The second strategy, termed entry testing*,* aimed at detecting the intrusion of the virus into the clinic at the entry point, in addition to baseline surveillance. To this end, patients which were newly admitted to the clinic or who returned from temporary absence were tested immediately when entering the clinic and five days after, which accounted for potentially long incubation times. The third and fourth strategy implemented regular testing of the clinic population on top of entry testing and baseline surveillance, i.e. testing every agent once weekly or twice weekly, respectively. These regular tests were simulated to be performed each Friday for once weekly testing and additionally each Tuesday for twice weekly testing. Considering that the result likely depends on slight variations in the incubation time or presymptomatic time for which exact knowledge about the distribution is sparse and constantly shifting, different testing days have not been analysed. As individual agents may refuse to participate in preventive testing measures, compliance to testing measures was defined as an additional agent property which was randomly assigned to each agent independent of their role with a certain probability given by the corresponding parameter. Non-compliant agents were not tested for strategies which imposed regular testing on the clinic population, i.e. for the third and fourth strategy. However, all agents were assumed to be compliant to isolation based on symptoms or a positive test result as this was the case in the actual clinic.

### Quantifying outcomes

In order to quantitatively evaluate the efficacy of the different strategies, suitable outcome measures were defined. The primary outcome measure of interest was the reduction of outbreak probabilities between two defined strategies. An outbreak was defined as $\mathrm{N}\ge 3$ new infections over a time span of $\mathrm{T}=10$ days, given a 100 day simulation run. The outbreak probability was defined as the proportion of simulation runs in which an outbreak occurred. Variations of parameter assumptions affect the outbreak probability for different strategies similarly. Consequently, ratios of outbreak probabilities of two strategies are likely less impacted by the parameter uncertainty than it is the case for absolute probability values. Stochastic uncertainty was minimized by generating at least 200.000 simulation runs for any simulation scenario, i.e. for any combination of parameters and strategy considered. The remaining stochastic uncertainty of results was indicated by error bars where applicable. In order to assess parameter uncertainty comprehensively, all model parameters were varied in a 1-way sensitivity analysis. This analysis varied one parameter at a time within its existing uncertainty, keeping all other parameters fixed at their best guess value. Parameters which have been observed to have little to no impact on results were excluded in the final analyses displayed here. In order to assess practicability of the proposed strategies, the amount of people under quarantine and the number of tests conducted per day were monitored as secondary outcomes.

## Results

### Quantifying outbreak probability reduction

**Fig. 3:**
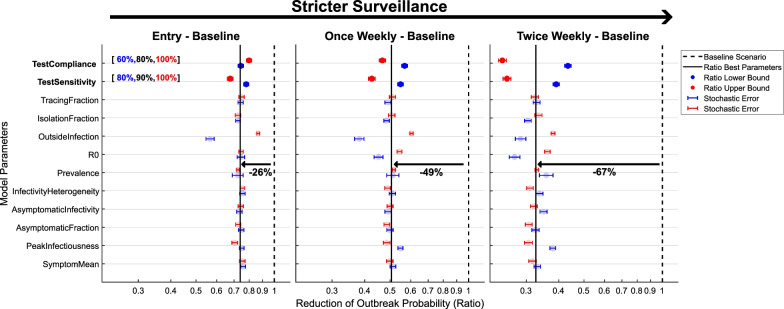
Reduction of outbreak probability by active testing strategies relative to the symptom-based baseline strategy. Results are illustrated on a log2-scale. The black lines correspond to the estimate of outbreak reduction for the best guess parameters. Each point corresponds to the estimated outbreak reduction for a 1-way sensitivity analysis of the corresponding parameter towards its upper bound (red) or lower bound (blue). Uncertainties due to stochasticity of the dynamics are visualized by 1$\sigma$ error bars. The results for the expected reduction of the outbreak probability are robust to most epidemiological parameter assumptions, the exact parameter values employed are stated in Table 1

The relative reduction of outbreak probability by entry, once weekly and twice weekly testing relative to the symptom-based baseline strategy is displayed in Fig. 3 for all parameter combinations relevant to the 1-way sensitivity analysis.

Implementing entry testing reduced the probability of an outbreak by 26% relative to the baseline only strategy, additionally testing of the clinic population once or twice weekly reduced the outbreak relative to the baseline strategy by 49% and 67%, respectively. The best guess outbreak probability reductions were mostly robust to variations of parameters and the large number of simulations conducted lead to narrow stochastic uncertainties. The sensitivity of the diagnostic test and compliance of the clinic population critically determined the efficacy of the strategy. Both of these parameters are not epidemiological quantities and can therefore be target for optimization in applications, which due to their large impact may lead to a considerable reduction in outbreaks. Epidemiological parameters with noticeable impact on the uncertainty of results were the reproduction number (R0), the probability of contracting an infection outside of the clinic (OutsideInfection) and the timing of the peak of infectiousness (PeakInfectiousness). An analysis of best-case and worst-case parameters for outbreak probability reduction provides a span of results of 8% to 61% for entry testing, 22% to 86% for once weekly testing and 37% to97% for twice weekly testing.

In order to assess the efficacy of the symptom-based baseline surveillance, it has been compared to a scenario with no surveillance in Additional file 3: Fig. S2 analogously to the analysis in Fig. 3. Conducting baseline surveillance compared to no surveillance at all reduced the probability of an outbreak by 49% assuming best guess parameters. This result lacked robustness to many parameter assumptions, ranging between values of 32 to 68%. Notably, this result was sensitive to assumptions on parameters which are important to the efficacy of symptom-based surveillance, such as the proportion of asymptomatic cases (AsymptomaticFraction), the timing of the peak of infectiousness (PeakInfectiousness), the reproduction number (R0) and the success rate of symptomatic screening (IsolationFraction).

### Test specificity drives practical feasibility

Practical feasibility of the strategies was assessed by analysing the secondary outcomes, i.e. the amount of tests conducted per day and the amount of individuals in quarantine on a given day. Since testing twice weekly was the most extensive testing strategy proposed, these outcomes have been analysed in a full 1-way sensitivity analysis. The results are visualized in Additional file 3: Fig. S3. For the best guess parameters, approximately 37 tests were conducted daily and one individual was isolated in quarantine per day in a clinic of approximately 140 individuals. The number of tests was predominantly determined by the compliance of the clinic population. The main driver of total quarantine time was the specificity of the diagnostic test. If the specificity of the test is decreased from 99.5 to 98%, the average amount of people in quarantine per day increased fourfold. This is reasonable since even in the absence of infections, false positives are expected regularly due to testing of the whole clinic population twice weekly. All other parameters had a comparably negligible effect, suggesting that effects such as over-sensitive symptom detection will likely not limit the practical applicability of surveillance. While test specificity does have a large impact on the average quarantine time, it is mostly irrelevant in preventing outbreaks as false positives are irrelevant for infection spread compared to false negatives.

### Effective surveillance requires immediate test results

The test-to-result delay of diagnostic tests differs between PCR and PoC antigen tests. Thus, analysing the effect of this delay on the efficacy of surveillance measures may provide valuable insight regarding their usefulness in application. In order to demonstrate possible effects, the different surveillance strategies were simulated for delays of ${\mathrm{t}}_{\mathrm{del}}\in \left[\mathrm{0,1},2\right]\mathrm{ days}$ in contrast to the previous analyses which assumed immediate availability of test results. Assuming previously demonstrated robustness of results, simulation runs were evaluated under the best guess parameters for all considered delays and all surveillance strategies.

**Fig. 4:**
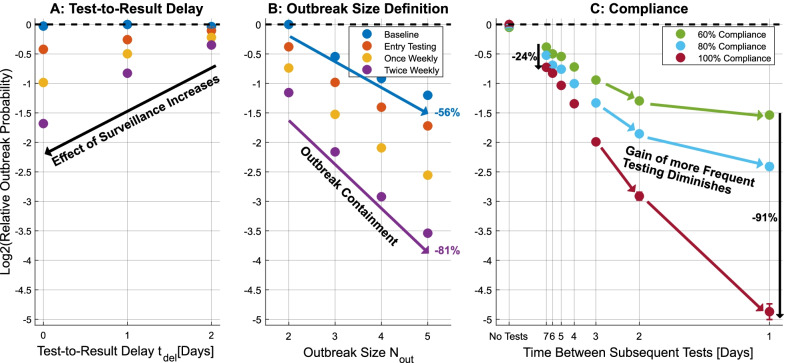
Impact of test-to-result delay (**A**), definition of outbreak size (**B**) and compliance (**C**) on the relative outbreak probability. The vertical axes denote outbreak probabilities on a log 2-scale, normalized relative to the largest outbreak probability of the respective analysis. Uncertainties due to stochasticity of the dynamics are visualized by 1$\sigma$ error bars, but these are mostly smaller than the point size. Results correspond to the best guess parameter set (except for changes for the particular analysis). **A** Decreasing test-to-result delay leads to more effective surveillance. **B** Decreasing probabilities within a strategy implies containment of ongoing outbreaks. **C** Different levels of compliance are analysed for various regular testing frequencies implemented on top of the symptom-based baseline surveillance strategy and entry testing. The horizontal axis corresponds to a frequency scale, as test frequency is proportional to test resources required. Benefits of increasing the test frequency are limited by lack of compliance, especially if test frequency is already high

Figure 4A shows the outbreak probabilities normalized relative to their value for baseline surveillance. The effect of increased surveillance was much more pronounced if the test-to-result delay is small, rendering the preventive effect of additional measures almost useless if the test-to-result delays reaches two days. The decrease in efficacy was likely due to the existence of symptomatic isolations in the baseline surveillance setting. Additional surveillance testing will only improve upon baseline surveillance if infected individuals are detected before they become symptomatic. In order to improve on a setting in which symptom-based baseline surveillance is practiced, diagnostic tests have to provide fast test results to conduct effective surveillance.

### Strict surveillance improves outbreak containment

Variation of the outbreak size, defined earlier as $\ge 3$ infected individuals over a course of 10 days, provides insight about the effective containment of ongoing outbreaks. To this end, outbreaks have been simulated for the four defined strategies assuming best guess parameters and the outbreak probability for outbreak sizes ${\mathrm{N}}_{\mathrm{out}}\in [\mathrm{2,3},\mathrm{4,5}]$ has been evaluated. Figure 4B shows the outbreak probabilities relative to their largest value, i.e. the baseline surveillance strategy with ${\mathrm{N}}_{\mathrm{out}}=2$. For each strategy, outbreaks of a larger size occur less frequently than smaller outbreaks. This implies that there is containment of outbreaks, i.e. there is a chance that outbreaks of a given size do not grow more severe and the outbreak is therefore stopped. More extensive surveillance strategies lead to a stronger reduction of the outbreak probability with increasing outbreak size, indicating that these strategies are more likely to completely cease ongoing outbreaks. While approximately 44% of outbreaks of size 2 evolved to outbreaks of size 5 for the baseline surveillance strategy, this fraction dropped to about 19% of outbreaks for testing twice weekly. Consequently, this analysis also implies that the benefit of implementing more active surveillance is even more pronounced in preventing larger outbreaks.

### Lack of compliance limits efficacy of regular testing

A population fully compliant to active surveillance testing is not guaranteed in an application setting. Therefore, the impact of varying levels of compliance with regular preventive testing measures in the clinic population was investigated. Different test frequencies were analysed using the best guess parameters and assuming established baseline surveillance and entry testing. Figure 4C shows the resulting outbreak probability for different frequencies and varying levels of compliance relative to the outbreak probability of the strategy without additional testing. Increasing compliance from 60 to 100% of the population decreased the outbreak probability by 24% if testing was conducted once weekly and by 91% if testing was conducted daily. Thus it is concluded that a lack of compliance limits the efficacy of regular testing, especially if testing frequency is high.

## Discussion

The ongoing COVID-19 pandemic necessitates appropriate public health responses to mitigate consequences for vulnerable populations. Facilities providing long-term treatment for psychiatric patients or care homes for elderly residents provide exemplary settings in which infection surveillance can be conducted at the level of a single institution. Diagnostic tests provide the means to establish surveillance, as they have been essential to detect ongoing outbreaks and prevent further spread of the virus and will continue to be at the center of infection control in the light of emerging virus mutations [33]. With growing accessibility of diagnostic tests, understanding their role in preventing outbreaks provides insight in how to conduct effective surveillance in an application setting.

We employed an individual-based model tailored to the setting of a typical mental health treatment facility to explore different surveillance strategies intended to suppress COVID-19 outbreaks. We modelled four surveillance strategies: symptom-based baseline surveillance, entry testing, testing once a week or testing twice a week. For each active surveillance strategy, estimates for the relative reduction of outbreak probability compared to the baseline strategy were obtained. We investigated critical determinants of the epidemiological dynamics and found that fast test results, high compliance and high test sensitivity are crucial to maximize efficacy of active surveillance. Investigation of the average number of diagnostic tests conducted and individuals under quarantine per day showed that regular testing is practically feasible if specificity of the diagnostic test is sufficiently high as this implies a low number of false positive test results.

A comprehensive sensitivity analysis confirmed the robustness of the obtained results under varying assumptions about a set of uncertain parameters, representing alternative epidemiological parameters as well as alternative structural assumptions. Moreover, the stochastic nature of the simulation model has been controlled for by generating a sufficient amount of simulation runs. Although an appropriate analysis of uncertainty has been conducted, the 1-way sensitivity analysis employed does not yield confidence or credible intervals on the generated estimates. However, the 1-way sensitivity analysis provides easily interpretable outcomes and introduces no additional bias compared to probabilistic sensitivity analyses, which require the specification of poorly known parameter probability distributions [25].

The best-case and worst-case ranges represent extreme case parameter sets and overestimate the underlying parameter uncertainty. While this can help to put results into perspective, the true effect estimates are much more likely to be in the vicinity of the reductions estimated for the best guess parameters. Additionally, some of the parameters with large impact on the model such as the test sensitivity and compliance are not necessarily unknown and should therefore be interpreted apart from truly uncertain epidemiological parameters, which is not considered in the best/worst case analysis.

We focused on the suppression rather than mitigation of outbreaks, defined here as 2–5 newly infected individuals over the course of 10 days. This approach differed from previous modelling studies which considered outcomes that focus on late outbreak stages, e.g. the number of cumulative infections [7–9]. The simulation of an outbreak for a long period of time amplifies the uncertainty in the assumed transmission mechanisms and in the epidemiological parameters due to the non-linear infection dynamics. Consequently, focusing the analysis on prevention of small outbreaks leads to uncertainties that can be controlled more realistically.

The small outbreak setting additionally reduces the impact of the modelled contact structure on the simulation results considerably. Indeed, we could see in our study that the impact of the parameter controlling the extent of heterogeneity in the contact structure was negligible. Therefore, only the variation of individual transmissibility needed to be incorporated into the model, but not necessarily an explicit contact structure as employed in many other studies [7–9, 31] including commonly encountered models with contact structure dictated by a network. This can be attributed to the fact that on average, the first few transmissions rely on the more general epidemiological parameters such as the reproduction number rather than the contact structure. Limiting results to small outbreak sizes is not restrictive in the practical application as even small outbreaks have major consequences for psychiatric clinics or skilled nursing facilities and should, therefore, be avoided as rigorously as possible.

Diagnostic tests have been characterized in the model by their sensitivity, specificity and their test-to-result delay without explicitly relating these to a PCR or PoC test. A PoC antigen test will usually imply a short test-to-result delay, while a PCR test should come with the benefit of a higher sensitivity and specificity. The value of these performance measures will vary between manufacturers, virus variants and the quality of the sample, such that the model results are to be interpreted in the light of the assumed test characteristics. While from a mathematical point of view it is legitimate to consider diagnostic tests with low sensitivities, surveillance with only low-sensitivity tests is unreasonable in the context of the case-ascertainment process assumed in the model. In applications, faster low-sensitivity tests can be used to screen for cases as long as results are confirmed with a diagnostic test with adequate sensitivity and specificity. This non-trivial model extension was not considered in our analyses. Diagnostic tests quickly become unsuitable for regular surveillance testing if their specificity is too low, because the resulting amount of false positives is problematic in active surveillance settings, which is well supported by our model results.

Another important structural limitation is the assumption of constant sensitivity of diagnostic tests during the course of disease. To account for this, the course of disease could be defined based on a viral load profile across time which is proportional to the infectiousness of an individual as well as to the sensitivity of the diagnostic test [30]. Current evidence suggests that PoC antigen tests can indeed perform well to detect relevant levels of viral load [24], highlighting the difficulty in establishing PoC test performance when compared to PCR tests as a reference standard [4]. In order to allow for a fair comparison between surveillance based on PCR tests and PoC antigen tests different detection threshold levels for viral load have to be included in the model. However, this does not affect our conclusion that short test-to-result delays are crucial for effective surveillance.

## Conclusions

COVID-19 surveillance in hospitals with long treatment duration and long-term care facilities provides a unique opportunity to create a safe environment for a vulnerable population. In this context, adequate assessment of the gain of various mitigation strategies is sparse but urgently needed to establish standards for practical implementation of strategies. In order to complement the existing literature, we quantified the effect of various surveillance strategies on the probability of occurring viral outbreaks. We demonstrated that implementing these strategies is practically feasible. We found that improving strategies based on isolating symptomatic individuals by means of testing-based strategies requires fast diagnostic test results, highlighting a possible use of point-of-care tests in this setting. Furthermore, we highlight the importance of a compliant population in order to maximize efficacy of regular testing. Overall, our results suggest that establishing surveillance exceeding symptom-based screening alone can successfully reduce disease burden in hospitals and long-term care facilities.

## Supplementary Information

**Additional file 1.** Summary of epidemiological characteristics for the article.

**Additional file 2.** Additional methods for the article.

**Additional file 3.** Additional results for the article.

## Data Availability

The MATLAB code used to generate the study results is available in a Zenodo repository, https://www.doi.org/10.5281/zenodo.4898924. The datasets used and/or analysed during the current study available from the corresponding author on reasonable request.

## Abbreviations

COVID-19: Coronavirus disease 2019
$$\mathrm{E}$$: Exposed
$${\mathrm{I}}_{\mathrm{A}}$$: Asymptomatically infectious
$${\mathrm{I}}_{\mathrm{P}}$$: Presymptomatically infectious
$${\mathrm{I}}_{\mathrm{S}}$$: Symptomatically infectious
$${\mathrm{N}}_{\mathrm{out}}$$: Outbreak size
PoC: Point-of-care
PCR: Polymerase chain reaction
$$\mathrm{R}$$: Recovered
R0: Reproduction number
$$\mathrm{S}$$: Susceptible
SARS-CoV-2: Severe acute respiratory syndrome coronavirus type 2
${\mathrm{t}}_{\mathrm{del}}$: Test-to-result delay
