# Supplementary material for: Preventing COVID-19 outbreaks through surveillance testing in healthcare facilities: a modelling study
Source: BMC Infect Dis. 2022 Jan 29;22:105. doi: 10.1186/s12879-022-07075-1 (PMC8800405; doi:10.1186/s12879-022-07075-1)
Supplement: Supplementary file 1 — Additional file 1. Summary of epidemiological characteristics for the article. [file 12879_2022_7075_MOESM1_ESM.docx]

Additional File 1: Summary of Epidemiological Characteristics for the article: *Preventing COVID-19 Outbreaks Through Surveillance Testing in Healthcare Facilities - A Modelling Study*

**Title:** Summary of Epidemiological Characteristics.

**Description:** Possible values of epidemiological parameters and their implications for the model have been discussed based on the existing evidence in the literature.

# Incubation Time

The incubation time is defined as time from infection to symptom onset. A meta-analysis across 11 studies [1] found a mean incubation time of 5.4 days with many studies being generally consistent with this value. An estimation of incubation time from case report data including time of exposure and manifestation of symptoms reported a lognormal distribution of incubation periods with a mean of 5.5 days with a 95%-IQR of [2.2,11.5] days, corresponding to a standard deviation of 2.4 days, although case data may underrepresent mild cases [2].

The incubation time is modelled as a log-normally distributed random variable with the assumption about the distribution adopted from [2]. This distribution is determined by its mean and its standard deviation which are extracted from the reported literature. Mean incubation time analysed in the model are reasonably certain and lie in the range $\mu_{inc}\in\left[ 5,6 \right] days$ and standard deviation lies in the range of $\sigma_{inc}\in\left[ 2.1,2.5 \right] days$ based on the 95% IQR reported in [2].

# Symptomatic Time

The symptomatic time as defined for this study is the time from symptom onset to the end of infectiousness. Information about this period can be obtained by various methodically different means. By monitoring the transmission history of index cases to their contacts, attack rates can be stratified by time after infection, providing insight into the time-dependent infectiousness. A study of this type concerned with infections after symptom onset in Taiwan found 12 transmission pairs with 11 infections occurring in the 1-3 day span after symptom onset and just 1 the 4-5 days span [3]. Early on in the pandemic, a study of 77 non-severe transmission pairs of Guangzhou hospital admissions modelled the infectivity profile explicitly and found that the infectiousness becomes vanishingly low 8 days after symptom onset [4]. Virological studies complement the body of evidence found by such contact studies by biological examination of patient samples. Viral RNA in these samples can be detected explicitly and investigated for their ability to reproduce, in which case the virus is likely still infectious. Detectable levels of viral RNA were reported to persist for 2 weeks and longer [5] and a scoping review reported that the time from symptom onset to two negative PCR-tests had a mean of 14 days across studies [6]. However, efforts to cultivate virus from patient samples failed for all tested samples after day 8 of symptom onset, [5, 7] suggesting that the highly sensitive detection of viral RNA is not a reliable proxy of patient infectiousness which is in accordance with reports of the scoping review [6]. Due to the technical difficulties of asserting active infectiousness, the overall evidence on the symptomatic time is scarce but suggests high infectivity around symptom onset and negligible levels of infectiousness for most individuals around day 8 after symptom onset.

The symptomatic time is modelled as a log-normally distributed random variable. Although information about the distribution could not be extracted from literature, the log-normal assumption is adopted as for the incubation time with parameters fitting the general descriptions in this paragraph. The mean symptomatic times are not entirely certain which is reflected in the large range of analysed parameters $\mu_{symp}\in\left[ 3.5,6.5 \right] days$ and the standard deviation is chosen to lie in the range of $\sigma_{inc}\in\left[ 1.1,1.9 \right] days$ to fit the available evidence.

# Presymptomatic Transmission

A characteristic feature of SARS-CoV-2 is a period of infectious viral shedding before symptoms manifest which is denoted as the presymptomatic time. The existence of presymptomatic transmission is reflected in a serial interval which is of a similar magnitude as the incubation time, implying that there must be significant presymptomatic transmission potential [8]. 2-point testing in a skilled nursing facility revealed that of 27 patients who were asymptomatic but SARS-CoV-2 positive at first test, 24 patients turned symptomatic with a median time of test to symptom onset time of 4 days, suggesting existence of viral RNA a couple days before symptoms appear [9]. In a study of transmission pairs, infectiousness was found to start from >2 days before symptom onset and relevant levels of infectiousness start 5 days before the onset of symptom. [4] A descriptive study of transmission clusters in Singapore found 10 presymptomatic transmissions which occurred 1-3 days before symptom onset [10]. A scoping review on infectious times found that most studies consistently report mean presymptomatic periods of 1-4 days [6].

The available evidence suggests that presymptomatic transmission is relevant and infectiousness can with some certainty be modelled to start in the range of 1-4 days before symptom onset. The presymptomatic time is modelled as a random variable which, due to lack of more specific information, is assumed to be uniformly distributed on the specified range of 1-4 days before symptom onset.

# Asymptomatic Transmission

Apart from the temporal properties of the natural history of disease, characteristics pertaining to asymptomatic individuals need to be included. The asymptomatic fraction, i.e. the fraction of infected cases who do not develop noticeable symptoms at any stage of the disease is of major importance when trying to predict how well different outbreak suppression strategies perform. With increasing numbers of scientific investigations in this regard, the existence of a large reservoir of completely asymptomatic individuals can be excluded. In an extensive meta-analysis, an asymptomatic fraction of 20% was reported across studies [11]. Specific studies with large numbers of participants which reported an asymptomatic fraction are the study on the Diamond Princess cruise ship [12] which reported 113 (18%) asymptomatic patients and a retrospective cohort study in Korea reporting 89 (29%) asymptomatic individuals [13]. In a screening study of healthcare workers in the UK [14], 6 completely asymptomatic individuals (11%) were identified, while this number climbed to 18 individuals (32%) if mild symptoms are counted as asymptomatic, providing a span of how symptom definition may affect the asymptomatic fraction. Although the definition of an asymptomatic case varies across studies, a general range of possible values can be specified.

In order to properly plan responses to the ongoing pandemic, the transmission potential of these asymptomatic carriers must be discussed. Cycle threshold (or $C_{t}$) values of a PCR test indicate the viral load available in the sample and they are defined as the number of reproduction cycles necessary to generate a detectable signal. Comparisons of $C_{t}$ values [9, 13] showed no significant difference in asymptomatic and symptomatic carriers. A review concerned with properties of asymptomatic carriers reported conflicting results in the literature concerning the existence of different transmission potential [15]. Another systematic review with a sample size of 5 studies found some evidence for a relative risk of infection smaller than 1 for asymptomatic carriers, but the confidence interval is consistent with 1 as well [11]. Consequently, the possibilities of asymptomatic infectiousness lower or equal to symptomatic infectiousness should both be considered.

Based on the available evidence, the fraction of cases $A$ which display no noticeable symptoms is modelled in the range of $A\in[0.1,0.3]$. Their infectiousness compared relative to symptomatic cases is denoted by $A_{rel}$ and takes values in the range of $A_{rel}\in[0.4,1]$.

# Reproduction Number

The most influential measure to classify the propensity of an infection to spread is the reproduction number $R_{0}$ which quantifies the average number of secondary infections emanating from a primary infection in a fully susceptible population. It is not an inherent characteristic of the pathogen but is instead highly contextual. To name a few factors, the behaviour of individuals, the current environment and protective measures like face masks or enhanced personal hygiene all impact the overall transmission rate and thus the reproduction number. To account for the highly contextual application, $R_{0}$ was varied across the wide range of $R_{0}\in[1.5,5]$ to reflect the existing uncertainty in this parameter in this specific setting.

# Generation Time

Assessing the effective virus propagation requires not only a reproduction number, but also the length of a typical reproduction cycle. This characteristic is covered by the generation time which is the time of the infection of a primary case to the infection of the secondary case. It is similar but not equivalent to the serial interval, which states the time difference between symptom onset of index and of secondary case. Unlike the generation time, the serial interval can also take negative values due to existence of presymptomatic transmission, but the mean can nevertheless be used as a proxy for the mean generation time. Generation times are a more fundamental measure for epidemiological characterizations, although also more complicated to infer. A study of cluster data in Singapore and in Tianjin found generation times of 5.2 days and 4.0 days respectively along with their respective distributions [16]. Serial interval estimation of publicly available data of 28 transmission pairs yielded a value of 4.7 days for the serial interval [8]. Interestingly, the study of transmission pairs in Guangzhou found that 8% of serial intervals are negative with an overall mean of 5.8 days [4]. Another study of 40 high confidence transmission pairs which directly estimated generation times found a mean generation time of 5.0 days as well as a corresponding distribution of times [17].

The generation time has not been used as a model input and can therefore be used to validate the infectivity profile to a limited extent. If infected individuals did not change their behaviour based on their disease state, the distribution of infectivity would be proportional to the distribution of generation times. Infectivity profiles can be randomly generated and thereupon generations times can be sampled to simulate the mean model generation time. This generation time estimate depends on several model parameters, i.e. the incubation time, the symptomatic time and the timing of peak infectiousness. In order to obtain the span of mean generation times which the model can realize, generation times are generated for the best parameter guess and a set of model parameters corresponding to both minimum and maximum mean generation time within the specified parameter uncertainty ranges. The minimal mean generation time realized by the model is 4.9 days, the best guess mean generation time is 6.4 days and the maximal mean generation time is 8.0 days. The stochastic error of these estimates is negligible compared to the rounding error. These generation times are on the large end compared to the generation times reported. But this is reasonably expected: Due to most individuals exhibiting symptoms at some point of their disease, they will tend to truncate the right side of their infectivity profile due to isolation. If this consideration is included in interpreting the simulation results, the generation times produced by the model fit well to the estimates in the literature.

# Peak Infectiousness

The time-dependent infectiousness was described in the main paper as linearly increasing at the start of the presymptomatic phase until a peak value is reached, after which it decreases linearly towards the end of the symptomatic phase. The timing of peak infectiousness has major implications for the spread of infection as it scales the infectivity of presymptomatic individuals: An early peak of infectiousness makes symptom-based isolation strategies less efficient. Peak infectivity is expected to occur around symptom onset, as the generation time is similar to the incubation time, implying common occurrence of presymptomatic transmission. This statement is supported by analysis of viral load dynamics in a scoping review of infectious periods, where viral loads were reported to peak on symptom onset or 2-4 days after [6].

The timing of peak infectiousness $t_{peak}$ is a model parameter which fixes the form of the infectivity profile. It is modelled in the range of $t_{del}\in\left[ -1,3 \right] days$ interpreted relative to the time of symptom onset. The possibility of infectiousness peaking before the onset of symptoms is therefore included.

Literature Cited

1. Zhang P, Wang T, Xie SX. Meta-analysis of several epidemic characteristics of COVID-19. Journal of Data Science 2020; 18(3):536–49.

2. Lauer SA, Grantz KH, Bi Q, Jones FK, Zheng Q, Meredith HR et al. The incubation period of coronavirus disease 2019 (COVID-19) from publicly reported confirmed cases: estimation and application. Ann Intern Med 2020; 172(9):577–82.

3. Cheng H-Y, Jian S-W, Liu D-P, Ng T-C, Huang W-T, Lin H-H. High transmissibility of COVID-19 near symptom onset 2020, medRxiv [Preprint]. Available from: URL: https://www.medrxiv.org/content/10.1101/2020.03.18.20034561v1, doi: 10.1101/2020.03.18.20034561.

4. He X, Lau EHY, Wu P, Deng X, Wang J, Hao X et al. Temporal dynamics in viral shedding and transmissibility of COVID-19. Nat Med 2020; 26(5):672–5.

5. Wölfel R, Corman VM, Guggemos W, Seilmaier M, Zange S, Müller MA et al. Virological assessment of hospitalized patients with COVID-2019. Nature 2020; 581(7809):465–9.

6. Byrne AW, McEvoy D, Collins AB, Hunt K, Casey M, Barber A et al. Inferred duration of infectious period of SARS-CoV-2: rapid scoping review and analysis of available evidence for asymptomatic and symptomatic COVID-19 cases. BMJ Open 2020; 10(8):e039856.

7. Bullard J, Dust K, Funk D, Strong JE, Alexander D, Garnett L et al. Predicting infectious SARS-CoV-2 from diagnostic samples. Clinical infectious diseases 2020; 71(10):2663–6.

8. Nishiura H, Linton NM, Akhmetzhanov AR. Serial interval of novel coronavirus (COVID-19) infections. Int J Infect Dis 2020; 93:284–6.

9. Arons MM, Hatfield KM, Reddy SC, Kimball A, James A, Jacobs JR et al. Presymptomatic SARS-CoV-2 infections and transmission in a skilled nursing facility. N Engl J Med 2020; 382(22):2081–90.

10. Wei WE, Li Z, Chiew CJ, Yong SE, Toh MP, Lee VJ. Presymptomatic transmission of SARS-CoV-2 - Singapore, January 23-March 16, 2020. MMWR Morb Mortal Wkly Rep 2020; 69(14):411–5.

11. Buitrago-Garcia D, Egli-Gany D, Counotte MJ, Hossmann S, Imeri H, Ipekci AM et al. Occurrence and transmission potential of asymptomatic and presymptomatic SARS-CoV-2 infections: A living systematic review and meta-analysis. PLoS Med 2020; 17(9):e1003346.

12. Mizumoto K, Kagaya K, Zarebski A, Chowell G. Estimating the asymptomatic proportion of coronavirus disease 2019 (COVID-19) cases on board the Diamond Princess cruise ship, Yokohama, Japan, 2020. Euro Surveill 2020; 25(10):pii=2000180.

13. Lee S, Kim T, Lee E, Lee C, Kim H, Rhee H et al. Clinical course and molecular viral shedding among asymptomatic and symptomatic patients with SARS-CoV-2 infection in a community treatment center in the republic of Korea. JAMA Intern Med 2020; 180(11):1447–52.

14. Rivett L, Sridhar S, Sparkes D, Routledge M, Jones NK, Forrest S et al. Screening of healthcare workers for SARS-CoV-2 highlights the role of asymptomatic carriage in COVID-19 transmission. Elife 2020; 9:e58728.

15. Nikolai LA, Meyer CG, Kremsner PG, Velavan TP. Asymptomatic SARS coronavirus 2 infection: invisible yet invincible. Int J Infect Dis 2020; 100:112–6.

16. Ganyani T, Kremer C, Chen D, Torneri A, Faes C, Wallinga J et al. Estimating the generation interval for coronavirus disease (COVID-19) based on symptom onset data, March 2020. Euro Surveill 2020; 25(17):pii=2000257.

17. Ferretti L, Wymant C, Kendall M, Zhao L, Nurtay A, Abeler-Dörner L et al. Quantifying SARS-CoV-2 transmission suggests epidemic control with digital contact tracing. Science 2020; 368(6491):eabb6936.
