# Supplementary material for: Preventing COVID-19 outbreaks through surveillance testing in healthcare facilities: a modelling study
Source: BMC Infect Dis. 2022 Jan 29;22:105. doi: 10.1186/s12879-022-07075-1 (PMC8800405; doi:10.1186/s12879-022-07075-1)
Supplement: Supplementary file 2 — Additional file 2. Additional methods for the article. [file 12879_2022_7075_MOESM2_ESM.docx]

Additional File 2: Additional Methods for the article: *Preventing COVID-19 Outbreaks Through Surveillance Testing in Healthcare Facilities - A Modelling Study*

**Title:** Supplementary Methods

**Description:** (1): Agent-based modelling. (2): Modelling Infection Dynamics. This section contains detailed information about the modelled mechanics of infection spread and model calibration. Figure S1: Individual infectivity and empirical offspring distribution. (3): Simulating Surveillance. This section describes the implementation of the surveillance aspects of the model. Table S1: Implemented agent properties. (4): Number of Simulations.

# Agent-based Model

Agent-based models provide features which deterministic models based on mean system behaviour such as SEIR differential equation models [1] or the Kermack-McKendrick formalism [2] cannot reproduce. In order to successfully model a system, the relevant features of its real-world counterpart need to be reflected in the model. This comprises i) the discreteness of the population as the system consists of only few agents, ii) the individual heterogeneity of these agents and iii) the inherent stochasticity of the underlying epidemiological dynamics. These aspects are generally not reproduced in deterministic approaches, but are inherent to agent-based simulation approaches. Such agent-based approaches constitute a flexible framework to model effects on the level of individual agents. Modelling on this level allows for specification of individual agent properties and explicit rules of how interaction and infection spread works in a highly customizable fashion [3]. Stochasticity of the epidemiological dynamics is readily incorporated by the stochastic simulation of the specified dynamics.

# Modelling Infection Dynamics

## Semi-Closed Environment

The infection dynamics of the system consists of two separate parts. Infection of agents outside of the clinic environment occurs by interaction with an environment with a certain number of effective contacts and some prevalence. This is in contrast to the interaction of individuals within the clinic which features a more detailed model of transmission based on the current state of infectious individuals currently present in the clinic. On each day of the simulation, agents are at risk of contracting the infection with a certain probability which is used to draw a Bernoulli random number signalling whether an infection actually occurred. If an agent becomes infected, they are assigned a random course of disease based on the epidemiological parameters discussed.

Mechanistically speaking, the probability of infection depends on the probability of contacting an infectious individual $P_{C}$ multiplied with the probability of infection transmission given an infectious contact $P_{I|C}$. These probabilities depend on agent properties, as $P_{I|C}$ is a function of the current infectivity of the corresponding agent and $P_{C}$ varies between different agents. Hence, for further discussion, these two parameters are defined as base rates and the mentioned modifications are subsequently introduced as modifiers for these base rates. In order to inform the intensity of infection spread within the clinic, the reproduction number $R_{0}$ is employed which is related to the product of these probabilities and can therefore only fix one of these two parameters. In order modify the infection risk outside of the clinic, another parameter is required in the model. When keeping $R_{0}$ fixed, the parameter $P_{I|C}$ can be used to modify the risk of infection outside of the clinic while keeping the risk of infection within the clinic fixed. The parameter $P_{I|C}$ scales the risk of infection with contacts outside of the clinic, while $R_{0}$ scales the risk of infection between clinic internal contacts.

The simulation of transmission is internally handled based on the parameters $P_{C}$ and $P_{I|C}$. Therefore, the probability of an agent being infected outside or inside the clinic is specified as a function of these parameters. This is discussed separately for the case of infection inside and outside the clinic.

## Infection Outside the Clinic

We define the probability of infection outside the clinic as the product

$$P_{inf}^{out}=P_{I|C}N_{c}P_{prev}$$

of the probability $P_{I|C}$ of getting infected given a contact with an infectious person, the expected number of close contacts $N_{C}$ and the local COVID-19 prevalence $P_{prev}$ in the population. The infection risk outside of the clinic is modified by the parameter $P_{I|C}$.

The expected number of close contacts has been guessed for the type of outside interaction, e.g. 0.2-1 close contacts for staff after their shift and 1-3 close contacts for patients when leaving the clinic over weekend. The exact values are generally not of critical importance, as the parameter $P_{I|C}$ (called OutsideInfection in Table 1) is explored in the range $P_{I|C}\in[0.01,0.16]$ in the conducted sensitivity analysis. However, the relative importance of different ways for the virus to intrude into the clinic affects the efficacy of entry testing, as workers after their shift are not tested contrary to patients returning from a temporary leave. This shall not be investigated further here as the exact values are hardly generalizable to other settings.

## Infection Inside the Clinic

### Calibrating $\boldsymbol{P}_{\boldsymbol{C}}$ by $\boldsymbol{R}_{\boldsymbol{0}}$

The reproduction number $R_{0}$ controls the intensity of infection transmission within the clinic and is treated as an external model input. Since the contact rate $P_{C}$ is required to calculate the probability of infection within the clinic, it needs to be calibrated by $R_{0}$. Employing the value for the transmission probability given a contact $P_{I|C}$, the base contact rate is calibrated to generate a pre-specified $R_{0}$. The exact equation on which the calibration is based looks as follows

| $R_{0}=P_{C}{\cdot P}_{I\vert C}\cdot N\cdot T\cdot(A\cdot A_{rel}+\left( 1-A \right))\cdot H$ | (1) |
| --- | --- |

or, equivalently, solved for the contact probability

| $P_{C}= \frac{R_{0}}{P_{I\vert C}\cdot N\cdot T\cdot(A\cdot A_{rel}+\left( 1-A \right))\cdot H}$ | (2) |
| --- | --- |

The variables used and the form of the equation are now subsequently explained and derived.

The probability of an infected individual transmitting the disease to any other agent in the clinic on a given day is $P_{C}P_{I|C}$, for $N$ agents in the clinic the expected number of infected individuals therefore is $R_{base}=NP_{C}P_{I|C}.$ This representation implicitly assumes that $N/{(N}-1) \approx1$ and it does not account for any modifications to infection probability, such as time-dependent infectivity profiles, less infectious asymptomatic agents and heterogeneous contact structure which are represented by $T, A, A_{rel}$ and $H$. The impact of these modifications on the reproduction number is now successively derived.

### Infectivity Profile

The time-dependent infectivity profiles of infected agents need to be accounted for when calibrating $P_{C}$. For each individual, a random *relative infectivity profile* with a peak normalized to 1 is drawn. The random *total relative infectivity* $\boldsymbol{T}$ is the sum over the relative infectivity of each day, represented by the area under the curve of the relative infectivity profile. By simulation of many values for $\boldsymbol{T}$, the expected total relative infectivity $T=E[\boldsymbol{T}$] can be estimated based on the generated sample. The reproduction number $R_{T}$ is the sum over all expected infections over the course of disease, therefore

| $R_{T}=P_{C}{\cdot P}_{I\vert C}\cdot N\cdot T$ | (3) |
| --- | --- |

represents the reproduction number if heterogeneity in contact structure and if infectivity of asymptomatic agents is ignored.

The retention times for different stages of disease are drawn from independent distributions such that some loose constraints are needed to enforce sensible courses of disease and infectivity profiles:

- The times are drawn from discrete distributions to comply with the time-discrete model structure.
- Incubation time and symptomatic time distributions are truncated to force them onto the interval [2 days, 15 days] with the small remaining mass located on the edges of the distribution.
- If the presymptomatic time exceeds the incubation time, infectivity starts at day 1 after infection.
- The peak of infectiousness can neither occur on day 1 of infectiousness nor on the last 2 days of infectiousness.

### Asymptomatic Infectivity

Asymptomatic agents might differ in their infectivity compared to symptomatic agents. In order to account for these differences, it is noted that

| $R_{TA}=A\cdot A_{rel}\cdot R_{T}+(1-A)\cdot1\cdot R_{T}$ | (4) |
| --- | --- |

represents the reproduction number which we provide as model input if heterogeneity of the transmission structure is neglected. The fraction of asymptomatic cases is denoted here by $A$ and their relative infectivity compared to symptomatic cases is denoted by $A_{rel}$. In this equation, $R_{T}$ represents the reproduction number associated with symptomatic cases.

### Heterogeneity in Transmission Structure

Heterogeneous interaction structure is introduced into the model by defining different classes of agents with varying amounts of contact between them. The four agent classes patients $P$, low-risk staff $S_{1}$, average-risk staff $S_{2}$, high-risk staff $S_{3}$ are considered as the determining factors in introducing the existing heterogeneity. The number of patients and staff is randomly drawn from the intervals $n_{pat}\in[50,60]$ and $n_{staff}\in\left[ 80,100 \right]$ and the three staff risk classes are populated with the proportions $[0.3,0.4,0.3]$.

An individual can be represented by an indicator vector ${V={(P,S}_{1},S_{2}, S_{3})}^{T}$ with zeros except for the correct identifier variable which is set to one. The intensity of transmission between two agents is scaled by two matrices:

$$M_{risk}=\left( \begin{matrix} 1 & 1/{h_{mod}} & 1 & h_{mod} \\ 1/{h_{mod}} & 1 & 1 & 1 \\ 1 & 1 & 1 & 1 \\ h_{mod} & 1 & 1 & 1 \end{matrix} \right)M_{cont}=\frac{1}{2}\left( \begin{matrix} 2 & 1 & 1 & 1 \\ 1 & 1 & 1 & 1 \\ 1 & 1 & 1 & 1 \\ 1 & 1 & 1 & 1 \end{matrix} \right)$$

$M_{risk}$ describes variations in the risk of transmission due to staff occupation and has a variable parameter $h_{mod}$ (HeterogeneityModifier) which is introduced on empirical basis to investigate possible effects of heterogeneity.$M_{cont}$ describes the basic contact structure between agents which reflects the limited time frame for possible patient-staff interactions compared to patient-patient interactions. The overall transmission modifier due to heterogeneous interaction between two agents is determined by the matrix

$${{(M}_{trans})}_{ij}={{(M}_{cont})}_{ij}{{(M}_{risk})}_{ij}.$$

The modification of transmission probability $c_{mod}$ from agent in state $V_{1}$ to agent in state $V_{2}$ is then given by the corresponding component of the matrix:

$$c_{mod}=V_{1}^{T}M_{trans}V_{2}$$

This modifier is multiplied with the corresponding base transmission rate to incorporate interaction heterogeneity into the model. The transmission matrix has not been informed by other sources, but sensitivity analyses of $h_{mod}$ suggests that interaction heterogeneity is of inferior importance compared to other uncertainties in the small outbreak size setting. For larger outbreak sizes, network effects are likely to be important but are not captured by the proposed model structure, hence large outbreaks were not considered as discussed in the main text.

Correcting the reproduction number equation for heterogeneity is necessary since the scale of values in the transmission matrix $M_{trans}$ is arbitrary. Thus, these values need to be normalized such that they do not change the intensity of infection transmission. The transmission matrix $M_{trans}$ determines the transmission probability between two different agents rather than two different classes. In order to quantify the number of interactions between two classes, a matrix $M_{scale}$ is defined which quantifies interaction probability on a level of classes. The composition of $M_{scale}$ is set to depend on the size of the different groups of agent classes, as, for example, there are more agents in the patient class than in the high-risk staff class. Let $f_{i}$ denote the fraction of individuals in the agent classes enumerated by $i=\left\{ 1,2,3,4 \right\}$, then define

$${{(M}_{scale})}_{ij}=f_{i}f_{j}$$

and note that $\sum_{i,j} f_{i}f_{j}=\sum_{i} f_{i}\sum_{j} f_{j}=1.$ Now define

$${{(M}_{mix})}_{ij}={{(M}_{scale})}_{ij}{{(M}_{trans})}_{ij}$$

where $M_{mix}$ is a measure which specifies how likely a transmission between two classes is. $M_{scale}$ introduces a guess about which class is likely the transmitting class, while $M_{trans}$ specifies to which class infection is likely spreading.

The matrix $M_{mix}$ is needed to define $H= \sum_{i,j} {{(M}_{mix})}_{ij}$ which is used to normalize the scale of the transmission matrix $M_{trans}$, such that it only introduces heterogeneity without changing the intensity of infection transmission. This normalization is related to the final reproduction number $R_{0}$ by virtue of

| $R_{0}=R_{TA}\cdot H$ | (5) |
| --- | --- |

Combining equations (3)-(5) yield relates the reproduction number $R_{0}$ to the contact probability $P_{C}$ by virtue of equation (1), such that the the contact probability is calibrated by equation (2).

### Probability of Infection

Now that $P_{C}$ can be expressed by specification of $R_{0}$ in equation (2), the probability of infection $P_{inf,i,t}^{in}$ for an agent of class $i$ on a given day $t$ can now be specified by

$$P_{inf,i,t}^{in}=1-\prod_{j} ({1-P}_{C}\cdot\left( M_{cont} \right)_{ij}\cdot P_{I|C}\cdot\left( M_{risk} \right)_{ij}\cdot I_{t,j})$$

where the product is carried out over every infectious individual $j$ in the clinic and $I_{t,i}$ denotes the relative infectiousness on day $t$ as determined by the infectivity profile of individual $j$, including a possible decrease of infectiousness if the agent is asymptomatic.

### Empirical Offspring Distribution

Correctness of the calibration procedure can be validated internally by empirically comparing the reproduction number generated by the model with its pre-specified values. The reproduction number can be extracted from the more general concept of an offspring distribution which corresponds to a random variable describing the number of secondary infections a primary infector causes over the course of their disease.

For SARS-CoV-2, this distribution is characteristically over-dispersed. In order to model this over-dispersion, the relative infectivity of individual agents is set to a Gamma-distributed random variable with a mean value of 1 and varying shape parameters $\beta$ (InfectivityHeterogeneity). The shape parameter $\beta$ is inversely proportional to the variance $\beta\propto1/{\sigma^{2}}$when keeping the mean fixed. Effectively, the shape parameter controls the amount of over-dispersion in the offspring distribution, from equal individual infectivity for $\beta\to\infty$ to arbitrary large variance in individual infectivity for $\beta\to0$. Consequently, the structural assumption of varying individual infectivity is now parametrized by the shape parameter and can be readily included in sensitivity analyses. A visualization of the family of distributions of individual infectivity is shown in Figure S1A.


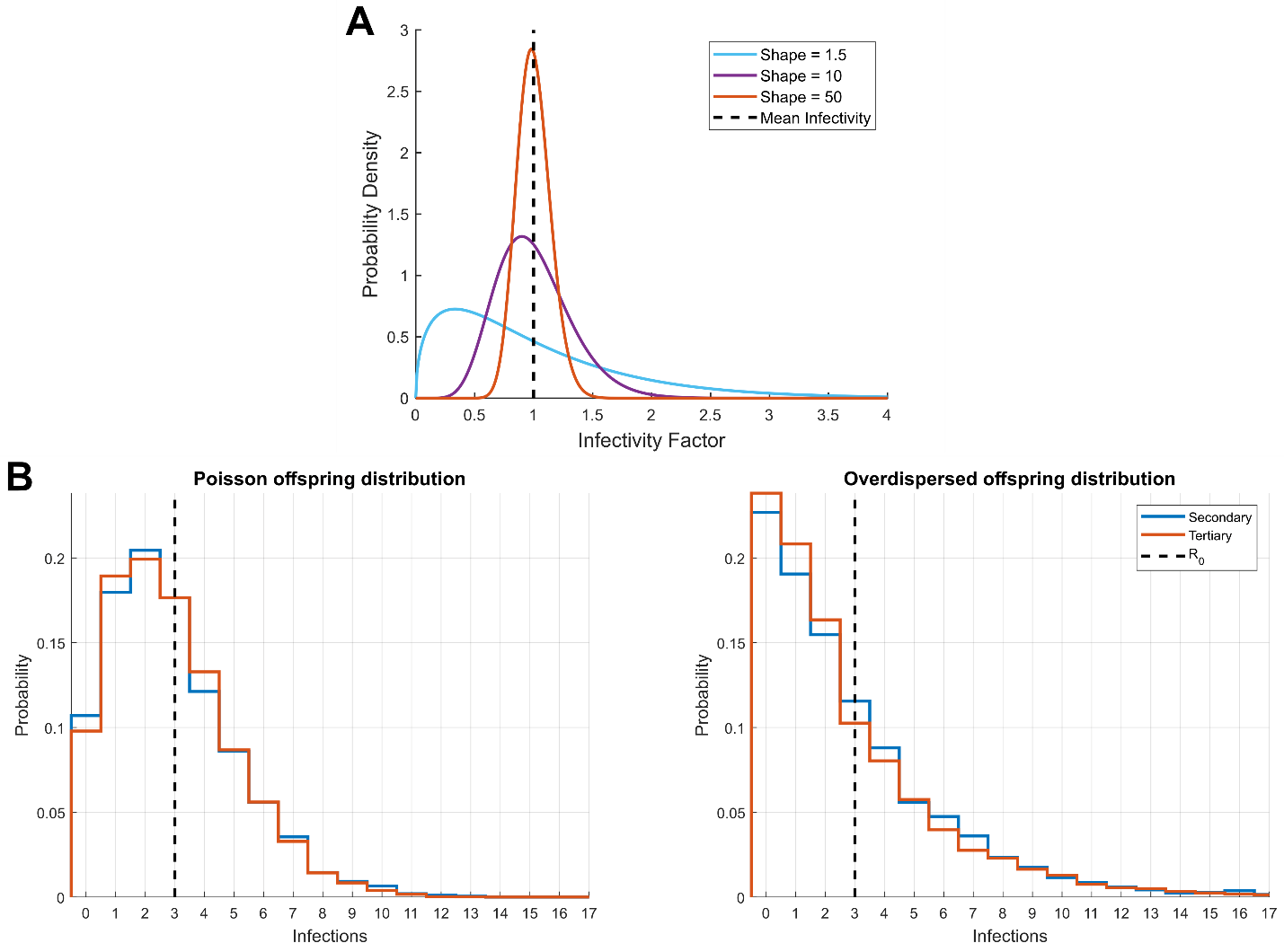


**Figure S1: Randomizing individual infectivity (A) and the impact on the offspring distribution of secondary cases (B).** **A:** Individual infectivity is sampled from gamma distributions with mean 1 and a variable variance which is increased by modifying the shape parameter while the mean is kept constant. The shape values were chosen for illustration purposes, the values used in the model are 1, 1.5 and 1000. **B:** Offspring distributions are empirically observed for the primary and secondary infectors in the model. If all individuals have the same infectivity, the offspring distribution is close to a Poisson distribution (left panel). Introducing individual heterogeneity with shape parameter 1.5 produces an over-dispersed distribution (right panel) which allows for more frequent occurrence of super-spreading.

Offspring distributions have been simulated by randomly seeding an infected case into the clinic and counting the number of secondary infections the index case causes. Additionally, the number of tertiary infections caused by each secondary infection is counted to assess consistency of results. There are some restrictions when counting the generated offspring to ensure that the whole course of disease is tracked which removes possibly occurring biases. Offspring distributions for homogeneous infectivity and the best guess estimate of individual infectivity are shown in Figure S1B in a simulation of unmitigated infection spread. The shape parameter of individual infectivity has been chosen to qualitatively resemble offspring distributions in empirical studies [4, 5]. It is observed that decreasing the shape parameter indeed increases the heterogeneity in the offspring distribution to a more reasonable extent. A more quantitative approach of specifying individual infectivity has been dismissed, because there are concerns with the limited amount of data and the extent to which this external data fits to the situation described by the model. However, these concerns pose no serious limitation as the uncertainty in the form of the offspring distribution is effectively addressed by sensitivity analyses.

Correctness of the model calibration is shown by the empirically generated offspring distribution in Figure S1B, as the empirical reproduction numbers of 2.92 and 2.93 for the secondary infections of both offspring distributions is close to the nominal value of $R_{0}$ = 3. This accuracy is acceptable when compared to the large range of values considered in sensitivity analyses.

# Simulating Surveillance

## Implementation of Dynamic Model

Agent properties and dynamic states provide the necessary information which feed into the simulation framework, providing the input to simulate the model dynamics. For each simulation run, the complete dynamic history of the simulation is stored, allowing for detailed analysis of the simulation if desired. The full list of agent-specific states and properties is provided in Table S1 combined with a description of the purpose of these variables. The model dynamics are simulated as informally described in the main text. For the complete description of the model dynamics, refer to the publicly available and documented code (<https://github.com/kreutz-lab/COVID19Surveillance>).

| **Name** | **Description** | **Purpose** |
| --- | --- | --- |
| ClassID | Defines agent class (patient/x-risk staff) | Calculate infection probability |
| Compliance | Binary indicator whether agents agree to regular testing | Indicates which individuals are tested in regular testing scenarios |
| DiseaseDay | Days since infection | Informs the current infectiousness and disease state |
| InfectionBy | Contains unique ID of infector if agent is infected | Information needed if contact tracing is initiated |
| InfectionCause | Source of infection (within clinic or which clinic external infection mode) | Additional analyses |
| InfectionDay | Simulation day of Infection | Reconstruction of infection dynamics |
| LastLeaveTimer | Days since patient entered the clinic (admission or temporary leave) | Indicates on which days agent is tested after entering the clinic (entry testing) |
| LeaveTimer | Days since agent temporarily left the clinic | Indicates when agent returns to the clinic |
| Presence | Binary indicator whether agent is present in clinic | Indicates whether outside or inside infection scheme is used for patients |
| QuarantineTimer | Days since isolation | Indicates when agents are re-tested in isolation |
| Quarantined | Binary indicator whether agent is under quarantine | Exclude quarantined individuals as a transmission risk |
| StartDay | Simulation day of patient admission | Regulates when patient are dismissed from the clinic |
| StateID | Disease state of individual | Informs many key components of the simulation dynamics. Contains additional state for permanently dismissed patients. |
| TestDelayTimer | Days since test was conducted | If test results are not available immediately, this counter regulates when test results can be accessed |
| TestResults | Result of last test | This variable is accessed once the test-to-result delay is over and signals isolation and contact tracing if result if positive |
| Tracing | Binary indicator whether agent has been affected by contact tracing | Regulation of tracing, such that only “first-order” tracing is employed. Resets after release from isolation. |
| UniqueID | Unique identifier of agent | Reconstruction of infection dynamics |
| Infectivity | Vector containing the relative infectivity of infected agent over time | Simulating infection dynamics |
| DiseaseCourse | Vector containing the different disease states of infected agent over time | Determines progression of disease states |

**Table S1: Dynamic and fixed agent properties as used in the implemented simulation model**. The “Purpose” section contains information about how these properties are utilized in the implemented model.

## Baseline Surveillance

All surveillance strategies conduct symptom-based isolations as well as contact tracing which was termed baseline surveillance. Symptom-based isolation is modelled as a random daily probability $p_{symp}$ (IsolationFraction) of successful isolation of individuals who are in the symptomatic disease state. On each day, a random Bernoulli draw with success probability $p_{symp}$ is performed for every symptomatic agent to simulate whether the agent is isolated or not. In order to include individuals which show symptoms of COVID-19 but are actually not infected with SARS-CoV-2, the possibility of unjustified isolations of symptom-based screening is considered. This is implemented by randomly drawing a number of such false isolations from a binomial distribution $\mathcal{B(}N,p_{symp}\cdot F_{symp}/N)$ where $F_{symp}$ (FalseSymptoms) is the expected number of daily false isolations under perfect isolation efficiency.

Contact tracing disrupts possible chains of infection once infected individuals are detected. Once contact tracing is initiated by a positive test, each infected contact is traced with a success probability $p_{trace}$ which is realized by random Bernoulli draws. In order to account for falsely traced individuals, the number of such agents is drawn from a binomial distribution $\mathcal{B(}N,p_{trace}\cdot F_{trace}/N)$ where $F_{trace}$ (FalseTraces) is the expected number of falsely traced contacts under perfect contact tracing efficiency. In both false symptomatic isolations and false contact tracing it has been implicitly assumed that the number of individuals isolated by mistake is proportional to the success probabilities of symptom-based screening and contact tracing respectively, as more strict measures should increase the number of erroneous alarms. Contact tracing is only conducted up to the first order, such that a positive test of a previously traced individual does not initiate another round of contact tracing.

## Active Strategies

Strategies involving active preventive testing of the clinic population are termed active strategies. The corresponding diagnostic test in the model is uniquely defined by its *sensitivity, specificity* and *test-to-result delay*. If an individual is in the presymptomatic, asymptomatic or symptomatic state, i.e. if the agent is infectious, the probability of a positive test result is assumed to be equal to its sensitivity. If the agent is in the susceptible, exposed or recovered state, i.e. non-infectious, the probability of a positive test result is chosen equal to 1-specificity. This implies that in our model the test accuracy is constant over the course of disease, disregarding changes in infectiousness. The test-to-result delay is defined as a lag between conducting the test and availability of its result, delaying possible countermeasures. Tested agents who are not under quarantine will isolate upon receiving a positive result, such that delays to this result lead to delays in isolation and initiation of contact tracing.

We defined the three active strategies *entry testing, once weekly* and *twice weekly* testing which define which individuals are tested on a given day. Additional analyses have been conducted to complement the results corresponding to the defined strategies, such as analysis of test-to-result delay, variation of outbreak sizes and the impact of compliance for various test frequencies. A summary of the different analyses is provided in Additional File 3: Table S2.

# Number of Simulations

The number of necessary simulations depends on the outcome of interest. A low prevalence of the disease in the environment outside of the clinic leads to a small number of outbreaks within the clinic. In general, a small number of events requires a larger number of simulation runs to generate an adequate sample size to calculate the probability of an outbreak. Let $x_{out}$ be the number of observed outbreaks in a sample of $N_{sim}$ simulations and let $p_{out}$ be the true outbreak probability for the simulation scenario, then we estimate the outbreak probability and its variance by

$${\overset{^}{p}}_{out}=\frac{x_{out}}{N_{sim}}, {\overset{^}{\sigma}}_{p_{out}}^{2}=\frac{{\overset{^}{p}}_{out}(1-{\overset{^}{p}}_{out})}{N_{sim}}.$$

The values reported in Figure 4 in the main text are based on the log2-probabilities, with the maximal value being shifted to zero. The new estimate for the variance follows by Gaussian error propagation:

$${\overset{^}{\sigma}}_{\log_{2}(p_{out})}^{2}=\log_{2}(e)^{2}\frac{{\overset{^}{\sigma}}_{p_{out}}^{2}}{{\overset{^}{p}}_{out}^{2}}$$

The error bars in the main text correspond to the square root of this estimated variance.

Outbreak probabilities between two strategies are compared as a log2-ratio of these probabilities. Effects of implementing surveillance are expressed as ratios because the efficacy of strategies is best expressed relative to each other. The desired interpretation of results is therefore that decreasing the outbreak probability from 20% to 10% or from 10% to 5% corresponds to the same relative effect size. This is true on a logarithmic scale, which is why all ratios are considered on a log2-scale. Additionally, ratios on a logarithmic scale are not affected by the bound at 0 for ratios on a non-logarithmic scale and hence are usually more appropriate to describe this type of data [6]. Denote the estimated outbreak probabilities of both scenarios by ${\overset{^}{p}}_{out}, {\overset{^}{q}}_{out}$. The error for the estimate of their log2-ratio $LR$ follows by Gaussian error propagation:

$${\overset{^}{\sigma}}_{LR}^{2}=\log_{2}(e)^{2}\cdot\left( \frac{{\overset{^}{\sigma}}_{p_{out}}^{2}}{{\overset{^}{p}}_{out}^{2}}+\frac{{\overset{^}{\sigma}}_{q_{out}}^{2}}{{\overset{^}{q}}_{out}^{2}} \right)$$

Error bars in Figure 3 correspond to the square root of the variance stated here. Considering log2-ratios of outbreak probabilities between scenarios increases the amount of simulation runs required compared to stating single outbreak probabilities.

The main issue of generating an adequately sized sample of simulations is the small frequency of events, i.e. the lack of outbreaks. A small event frequency originates from the small introduction rate of infected individuals into the clinic and effective surveillance strategies which detect individuals before they cause an outbreak. All our analyses related to outbreak probability or ratios of probabilities are therefore based on a sample size of at least 200.000 simulations (see Additional File 3: Table S2) in order to generate acceptably sized stochastic errors.

Literature Cited

1. Li R, Pei S, Chen B, Song Y, Zhang T, Yang W et al. Substantial undocumented infection facilitates the rapid dissemination of novel coronavirus (SARS-CoV-2). Science 2020; 368(6490):489–93.

2. Ferretti L, Wymant C, Kendall M, Zhao L, Nurtay A, Abeler-Dörner L et al. Quantifying SARS-CoV-2 transmission suggests epidemic control with digital contact tracing. Science 2020; 368(6491):eabb6936.

3. Pitman R, Fisman D, Zaric GS, Postma M, Kretzschmar M, Edmunds J et al. Dynamic transmission modeling: a report of the ISPOR-SMDM Modeling Good Research Practices Task Force-5. Value Health 2012; 15(6):828–34.

4. Hasan A, Susanto H, Kasim MF, Nuraini N, Lestari B, Triany D et al. Superspreading in early transmissions of COVID-19 in Indonesia. Sci Rep 2020; 10(1):22386.

5. Endo A, Abbott S, Kucharski AJ, Funk S. Estimating the overdispersion in COVID-19 transmission using outbreak sizes outside China. Wellcome Open Research 2020; 5:67.

6. Limpert E, Stahel, Werner, A., Abbt M. Log-normal Distributions across the Sciences: Keys and Clues. BioScience 2001; 51(5):341.
