# Supplementary material for: Preventing COVID-19 outbreaks through surveillance testing in healthcare facilities: a modelling study
Source: BMC Infect Dis. 2022 Jan 29;22:105. doi: 10.1186/s12879-022-07075-1 (PMC8800405; doi:10.1186/s12879-022-07075-1)
Supplement: Supplementary file 3 — Additional file 3. Additional results for the article. [file 12879_2022_7075_MOESM3_ESM.docx]

Additional File 3: Additional Results for the article: *Preventing COVID-19 Outbreaks Through Surveillance Testing in Healthcare Facilities - A Modelling Study*

**Title:** Additional Results

**Description:** (1): Table S2: Summary of analyses. (2): Figure S2: Comparison of baseline surveillance to no surveillance. (3): Figure S3: Analysis of secondary outcomes. (4): Figure S4: Absolute outbreak probabilities. (5): Figure S5: Comparison of active strategies to baseline surveillance for outbreak size $N_{out}=5$. (6): Discussion of entry testing. (7): Figure S6: Analysis of different entry testing days and test sensitivites.

# Introduction

This document lists additional results which provide more insight into the results of the main text. An overview of all the conducted analyses and their properties is provided in Table S2.

| **Analysis** | **Figure ID** | $\boldsymbol{N}_{\boldsymbol{sim}}$ | $\boldsymbol{t}_{\boldsymbol{del}}$ **[d]** | **Compliance** | $\boldsymbol{N}_{\boldsymbol{out}}$ | **Test Frequency** |
| --- | --- | --- | --- | --- | --- | --- |
| 4 Strategies ($\boldsymbol{N}_{\boldsymbol{out}}=\mathbf{3})$ | 3, S4 | 200.000 | 0 | [0.6,0.8,1] | 3 | [-,-,1x weekly,2x weekly] |
| Test-Delay | 4A | 200.000 | [0,1,2] | 0.8 | 3 | [-,-,1x weekly,2x weekly] |
| Outbreak Size | 4B | 2.400.000 | 0 | 0.8 | [2,3,4,5] | [-,-,1x weekly,2x weekly] |
| Frequency/  Compliance | 4C | 200.000 | 0 | [0.6,0.8,1] | 3 | Every [1,2,3,4,5,6,7,$\infty$] days |
| Quarantine/  Tests | S3 | 10.000 | 0 | [0.6,0.8,1] | 3 | 2x weekly |
| Baseline –  No Surveillance | S2 | 200.000 | 0 | - | [3,5] | Never |
| 4 Strategies ($\boldsymbol{N}_{\boldsymbol{out}}=\mathbf{5})$ | S5 | 200.000 | 0 | [0.6,0.8,1] | 5 | [-,-,1xweekly,2xweekly] |
| Entry Test Days | S6 | 200.000 | 0 | 0.8 | 3 | [-,-] |
| Different Test Sensitivities | S6 | 200.000 | 0 | 0.8 | 3 | [-,-,1xweekly,2xweekly] |

**Table S2: Brief summary of the properties of different model analyses.** $N_{sim}$: Number of simulations per point in result figures, $t_{del}$: Test-to-result delay, $N_{out}$: Outbreak size definition.

# Comparison of Baseline Surveillance to No Surveillance


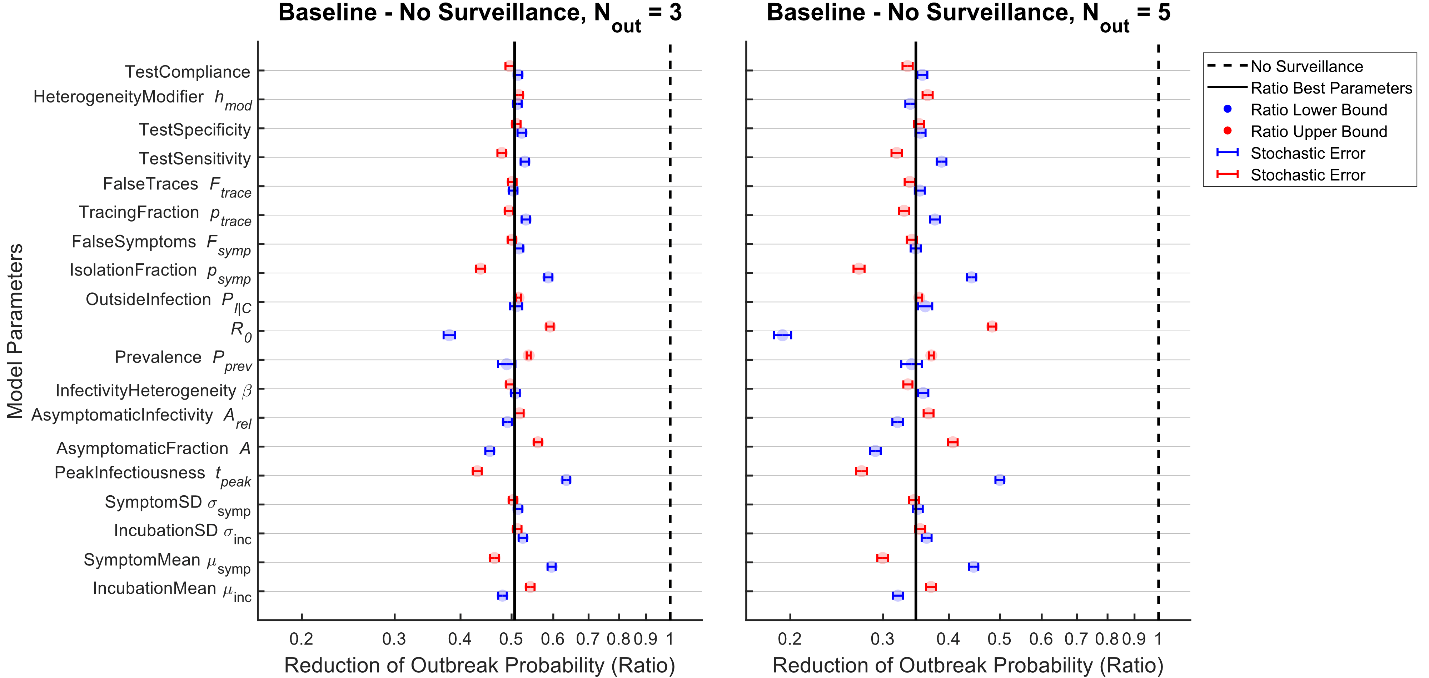


**Figure S2: Reduction of outbreak probability for the symptom-based baseline strategy compared to no surveillance for two outbreak sizes** $\boldsymbol{N}_{\boldsymbol{out}}$**.** Results are illustrated on a log2-scale. The estimate for the reduction of outbreak probability lacks robustness to many epidemiological parameters, in contrast to the main analysis in Figure 3. The effect size is sensitive to parameters which determine the efficacy of symptom-based surveillance, including the proportion of asymptomatic cases (AsymptomaticFraction), the timing of the peak of infectiousness (PeakInfectiousness), the reproduction number (R0) and the success rate of symptomatic screening (IsolationFraction). The impact of parameter uncertainty on the estimated reduction increases considerably when larger outbreak sizes are analysed.

# Analysis of Secondary Outcomes


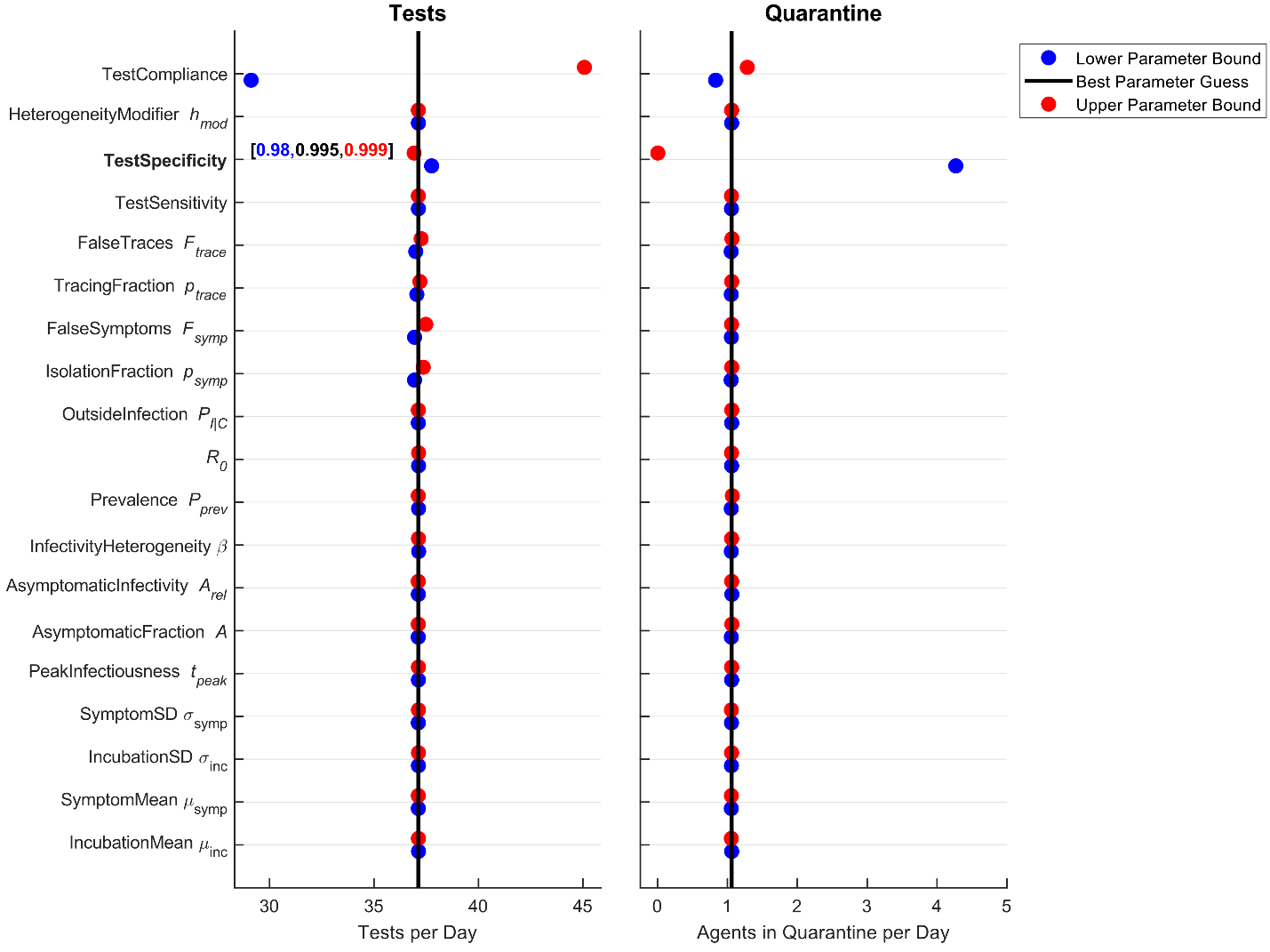


**Figure S3: Average number of tests conducted and average number of agents in quarantine per day for the strategy of testing twice weekly.** Stochastic errors are small and can be neglected. The daily number of tests does only drastically changes with varying test compliance. Quarantine time is sensitive to the specificity of the diagnostic test.

# Absolute Outbreak Probabilities

In the main text, the efficacy of different surveillance strategies has been quantified by relative reductions of outbreak probability between strategies rather than by absolute outbreak probability values. Figure S4 shows the absolute outbreak probabilities on which the relative reductions in Figure 3 in the main text are based on. Absolute outbreak probabilities are sensitive to parameter uncertainties, complicating the comparison between the different strategies. A strong correlation of changes in parameter and changes in outbreak probability across different scenarios is observed in this analysis. Therefore, a paired analysis considering ratios between outbreak probabilities for the same set of parameters is evaluated in the main text which is more robust against parameter uncertainties than absolute probability values.


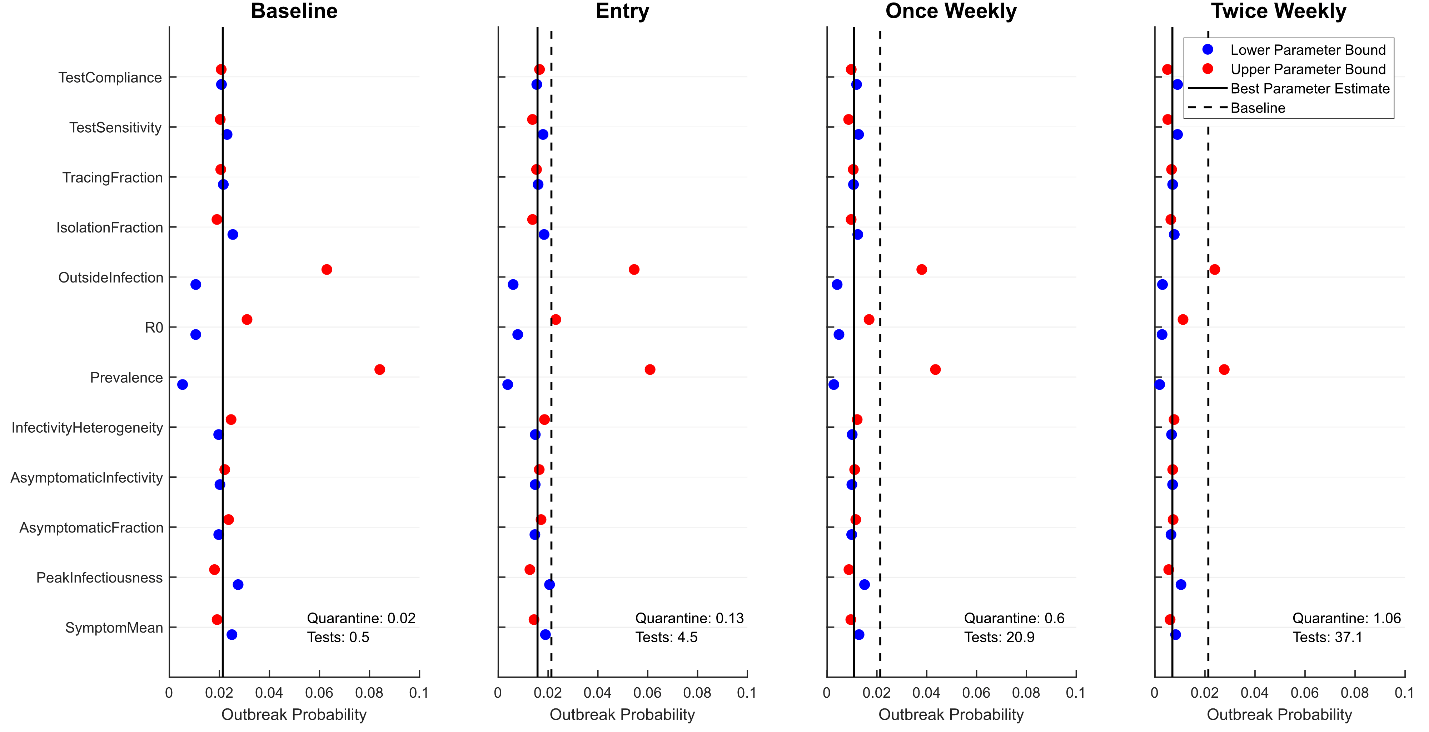


**Figure S4: Absolute outbreak probabilities for the analyzed strategies.** “Quarantine” denotes the average amount of individuals under quarantine per day and “Tests” denotes the average amount of tests conducted per day. Stochastic errors are smaller than the point size. The difference between the blue and red dots indicate the impact of parameter uncertainty: Absolute probabilities are sensitive to parameter variations, in particular with respect to R0, the prevalence and the probability of contact with an infected individual outside the clinic (denoted as OutsideInfection).

# Variation of the Outbreak Size

An outbreak has been defined as 3 infections over the course of 10 days in most analyses in the main text. Deviation from this definition is possible by varying the amount of infections which are counted as an outbreak.

Figure S5 shows the sensitivity analysis for the comparison of the three active strategies against the symptom-based baseline strategy for an outbreak size of $N_{out}=5$. Results are less robust to parameter uncertainty than for the outbreak size $N_{out}=3$ visualized in Figure 3 in the main text. Variation of the outbreak size effectively determines for how long the infection dynamics are monitored before an outbreak according to the previous definition has occurred. Simulation of the dynamics until the observation of a large outbreak amplifies the effect of existing parameter uncertainty on the outcome. This suggests that outcome measures linked to late outbreak stages might become unreliable. To further analyse the impact of uncertainty on results, consider the relative reduction of active testing compared to the baseline strategy in Figure S5 and the relative reduction of the baseline strategy to no surveillance in Figure S2. Comparing both figures reveals that the comparison of active strategy and baseline surveillance allows for conclusions much more robust to parameter uncertainties than the comparison of baseline surveillance to no surveillance.


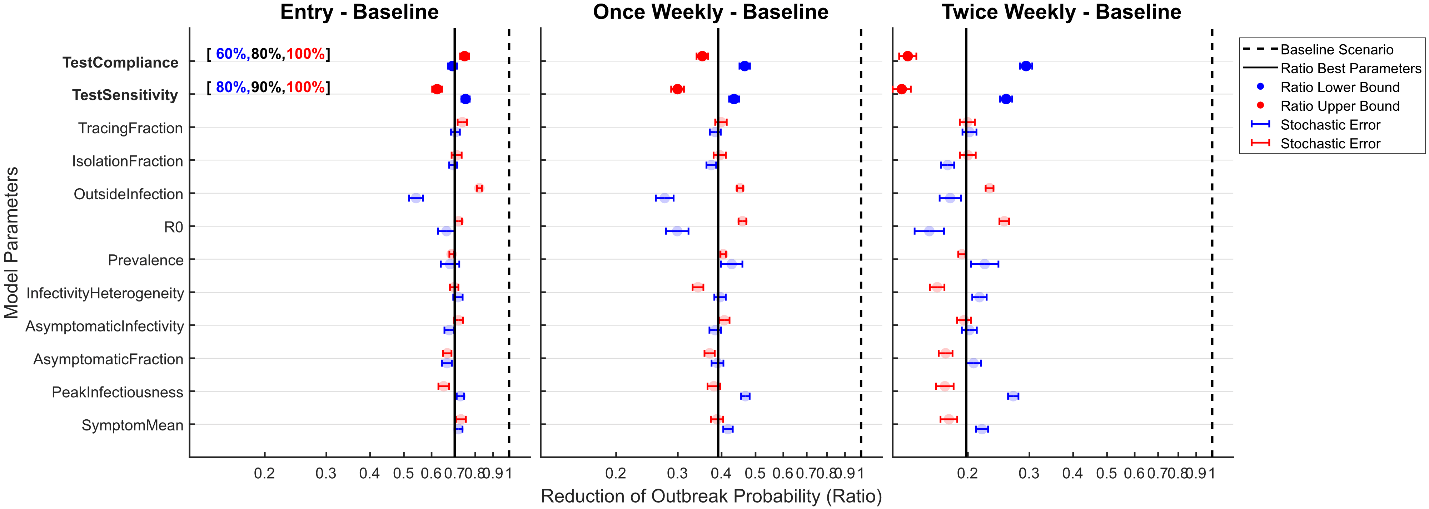


**Figure S5: Reductions of the outbreak probabilities by entry testing, once weekly and twice weekly testing relative to the baseline strategy for the outbreak size** $\boldsymbol{N}_{\boldsymbol{out}}\mathbf{=5}$**.** Results are illustrated on a log2-scale. The impact of parameter uncertainty is more pronounced in this sensitivity analysis than it was the case for the analogous analysis for outbreak size $N_{out}=3$ in Figure 3.

# Efficacy of Entry Testing

The potential reduction of outbreak probability due to entry testing significantly depends on the parameter OutsideInfection as observed in the sensitivity analysis in Figure 3 and Figure S5. The parameter OutsideInfection is proportional to the probability of an agent getting infected outside of the clinic. This is also true for the prevalence, but outcomes are not sensitive to this model parameter. In order to understand this discrepancy in the efficacy of entry testing, it is helpful to subdivide the infection influx into the clinic into three contributions:

1. Infection influx proportional to prevalence as well as OutsideInfection and reduced by entry testing (temporary leave of agents), denoted by $PO^{red}$
2. Infection influx proportional to prevalence as well as OutsideInfection but irreducible by entry testing (staff going home between shifts), denoted by $PO^{irr}$
3. Infection influx proportional to prevalence only and reduced by entry testing (patient admission), denoted by $P^{red}$

All three contributions to the infection influx into the clinic are proportional to the prevalence. Therefore, the relative reduction of cases due to entry testing is independent of the prevalence, as the fraction of cases detected by the measure is constant. The relative contribution of the irreducible component ${PO}^{irr}$ in the total infection influx is not independent of the parameter OutsideInfection: If this parameter is increased, $P^{red}$ makes up a smaller fraction of the total infection influx while the relative contribution of ${PO}^{irr}$ grows. As the relative contribution of the irreducible component ${PO}^{irr}$ to the total infection influx increases, the relative reduction of cases due to entry testing becomes smaller. This highlights the fact that the efficacy of entry testing necessarily depends on the relative contribution of cases which cannot be prevented at the entry point, i.e. by staff leaving the clinic between shifts.

# Changing Assumptions about Testing

The impact of a set of various testing strategies and assumptions about the performance of the diagnostic test has been analysed in the main text. The assumption about the timing of testing, e.g. testing individuals immediately on arrival in the clinic and five days after for entry testing, has briefly been discussed for the regular testing strategies, but not further analysed. Similarly, the impact of the three different test sensitivities 80%, 90% and 100% on the relative reduction between test strategies is illustrated in Figure 3 and choosing sensitivity values on the higher end has been discussed, but the impact of lower sensitivities has not been quantitatively analysed.


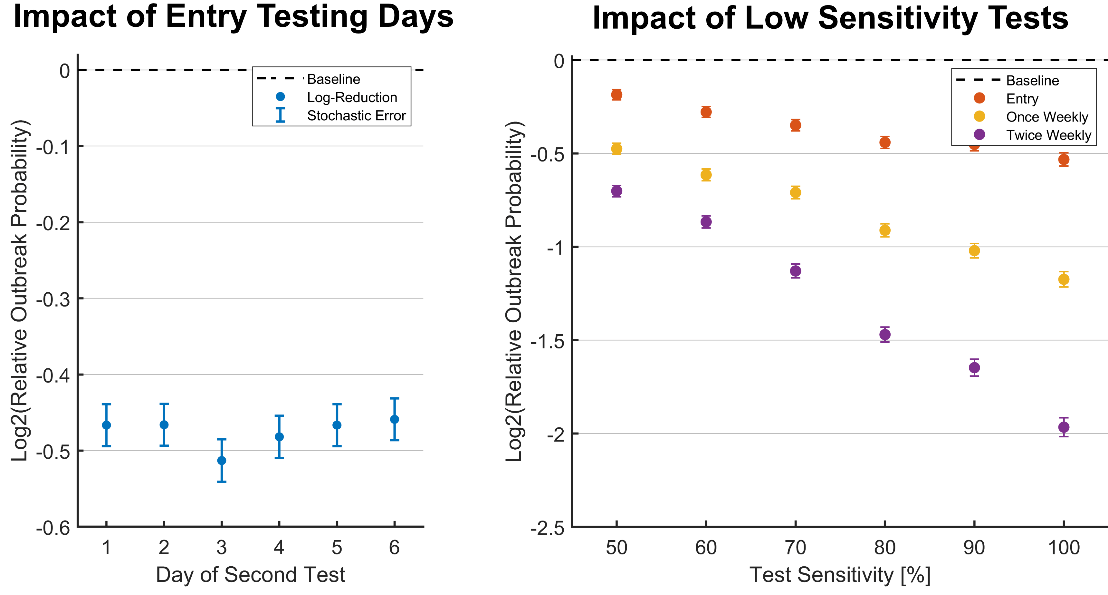


**Figure S6: The impact of different days for the second test in the entry testing setting (left panel) and and the impact of lower test sensitivities on the relative reduction of outbreak probability for the different strategies (right panel).** Results for the reduction relative to baseline surveillance are illustrated on a log2-scale and analyses are conducted for baseline parameters. **Left:** Choosing a different day for the second entry test may have a small impact on the performance of the strategy within the values used for this analysis, but the effect can also be explained by stochastic errors. **Right:** Employing low sensitivity tests for surveillance considerably reduces the impact of the different surveillance strategies.

Figure S6 shows results for these two quantitative analyses supporting the main results. The left panel illustrates the efficacy of entry testing for various possible days for a second test, after testing immediately on day zero of arrival. According to this analysis, testing on day 3 provides the largest decrease in outbreak probability, although the effect is small in any case and might be explained by stochastic errors. As discussed in the main text, this result will also be sensitive to assumptions about the incubation time as well as the presymptomatic time, such that caution is advised when interpreting this result. The right panel provides more insight about the efficacy of the different surveillance strategies under various assumptions about the test sensitivity, which are much lower than assumed in the main text. With decreasing test sensitivities, the relative reduction of the outbreak probability by the different testing strategies decreases considerably. While testing twice weekly reduces the outbreak probability by 40% compared to baseline if a test with 50% sensitivity is used, the reduction is about 75% compared to baseline if the diagnostic test used has 100% sensitivity. As discussed in the main text, surveillance with low sensitivity tests only is inappropriate if these tests additionally serve the purpose of case-ascertainment as assumed in the model.
